# Phytocompounds and Nanoformulations for Anticancer Therapy: A Review

**DOI:** 10.3390/molecules29163784

**Published:** 2024-08-09

**Authors:** Giuseppina Bozzuto, Annarica Calcabrini, Marisa Colone, Maria Condello, Maria Luisa Dupuis, Evelin Pellegrini, Annarita Stringaro

**Affiliations:** 1 National Center for Drug Research and Evaluation, Italian National Institute of Health, 00161 Rome, Italy; giuseppina.bozzuto@iss.it (G.B.); marisa.colone@iss.it (M.C.); maria.condello@iss.it (M.C.); marialuisa.dupuis@iss.it (M.L.D.); annarita.stringaro@iss.it (A.S.); 2 Laboratory of Experimental Oncology, IRCCS Istituto Ortopedico Rizzoli, 40136 Bologna, Italy; evelin.pellegrini@ior.it

**Keywords:** natural products, phytocompounds, nanoformulations, nanocarriers, drug delivery, nanomedicine, human cancer, drug resistance, anticancer therapy, combined therapies

## Abstract

Cancer is a complex disease that affects millions of people and remains a major public health problem worldwide. Conventional cancer treatments, including surgery, chemotherapy, immunotherapy, and radiotherapy, have limited achievements and multiple drawbacks, among which are healthy tissue damage and multidrug-resistant phenotype onset. Increasing evidence shows that many plants’ natural products, as well as their bioactive compounds, have promising anticancer activity and exhibit minimal toxicity compared to conventional anticancer drugs. However, their widespread use in cancer therapy is severely restricted by limitations in terms of their water solubility, absorption, lack of stability, bioavailability, and selective targeting. The use of nanoformulations for plants’ natural product transportation and delivery could be helpful in overcoming these limitations, thus enhancing their therapeutic efficacy and providing the basis for improved anticancer treatment strategies. The present review is aimed at providing an update on some phytocompounds (curcumin, resveratrol, quercetin, and cannabinoids, among others) and their main nanoformulations showing antitumor activities, both in vitro and in vivo, against such different human cancer types as breast and colorectal cancer, lymphomas, malignant melanoma, glioblastoma multiforme, and osteosarcoma. The intracellular pathways underlying phytocompound anticancer activity and the main advantages of nanoformulation employment are also examined. Finally, this review critically analyzes the research gaps and limitations causing the limited success of phytocompounds’ and nanoformulations’ clinical translation.

## 1. Introduction

In February 2024, the World Health Organization (WHO)’s cancer agency and the International Agency for Research on Cancer (IARC) released the latest estimates of the global burden of cancer on the basis of the best sources of data available in countries in 2022. Based on these data, there were about 20 million new cancer cases and 9.7 million deaths. Accordingly, approximately one in five people develop cancer in their lifetime, with a higher mortality in men (one in nine) than in women (one in twelve). Unfortunately, over the next 25 years, new cancer cases are expected to increase by 77% compared to the estimated 20 million cases in 2022 [1].

Cancer comprises a wide diversity of diseases, all of them characterized by numerous cellular physiological systems leading to abnormal and non-stop cell growth in a specific tissue location, forming a tumor and invading nearby tissues and organs, thereby interfering with normal body functions. Its complexity and multifactorial nature have always drawn great attention in the pharmaceutical industry and academic research [2].

Combined therapies are commonly used in current standards of care, applying different modalities such as surgery followed by chemotherapy and/or radiotherapy. Unfortunately, despite the advances in conventional treatment options, cancer therapy is still far from optimal [3]. Radiotherapy and chemotherapy are known for their significant adverse effects, with most methods non-specifically targeting any rapidly dividing cells without discriminating healthy from tumor tissues [3,4]. This lack of specificity results in the occurrence of many severe undesirable side effects and the problem of multiple drug resistance (MDR) [5].

MDR refers to the ability of tumor cells to gain resistance against a series of chemotherapy drugs with different structures and mechanisms [6]. Cancer cells can display an “intrinsic” resistance resulting from the constitutive overexpression of cancer cell detoxification systems that existed before the start of chemotherapeutic treatment, or they can acquire resistance resulting from mutations during the process of carcinogenesis or after the start of the chemotherapy over time. The MDR phenomenon is dependent on multiple pathways, including elevated metabolism of xenobiotics, enhanced efflux of drugs, growth factors, mutation and alteration in DNA repair pathways, defect in apoptotic pathways, autophagy, genetic factors (gene mutations, amplifications, and epigenetic alterations), the presence of cancer stem cells (CSCs), alterations in enzymes responsible for drug processing and metabolism, alterations in the composition of the cell membrane, modifications in the tumor environment, angiogenesis, and epithelial–mesenchymal transition [7].

Moreover, the poor pharmacokinetic characteristics of most anticancer drugs, arising from low solubility, stability, and metabolism and limited bio-distribution, have limited their clinical effectiveness. Thus, it is imperative to discover new drugs and develop effective formulations that can provide selective targeting of tumor sites without significant damage to healthy tissue viability [8,9,10].

Natural agents (also known as natural products), over the years, have raised great interest in the research and pharmaceutical industry as a source of new anticancer drugs, etc. As is well known, different forms of natural agents (e.g., oils, potions, remedies, and traditional medicines) have been used to care for various diseases and injuries [11]. Their medicinal properties then drew attention to the identification of bioactive compounds of interest, causing natural products to become valuable sources for drug discovery in various therapeutic areas, especially in the fields of oncology and infectious diseases, with the aim of using them as replacements for synthetic drugs.

For instance, most of the clinically available anticancer agents are plant-based bioactive products (phytocompounds) produced by plants for their protection [12]. Of these drugs, paclitaxel from the needles and bark of the Pacific yew tree *Taxus brevifolia*, vinca alkaloids vincristine and vinblastine from the Madagascar periwinkle *Catharanthus roseus*, etoposide from *Podophyllum peltatum*, and topotecan and irinotecan from *Camptotheca acuminata* represent some of the most clinically effective chemotherapeutic agents [13].

In addition to these, numerous cell culture studies and animal model systems have demonstrated that structurally different phytocompounds have cancer chemopreventive as well as chemotherapeutic activities [12,14]. These compounds can be classified into various structural classes, including alkaloids, terpenoids, organosulfur compounds, and phenolic compounds [15]. These plant-derived agents have a wide range of antitumor properties, including antioxidant, antiproliferative, proapoptotic, antiangiogenic, antimetastatic, and anti-inflammatory activities [11,12].

Despite the many positive health effects, the clinical effectiveness of phytocompounds is still limited due to their low absorption, poor solubility, low bioavailability, short retention time in biological systems, and the possible induction of cytotoxic effects. Moreover, after administration, phytocompounds encounter various biological, chemical, and physical barriers that can cause a change in their natural structure consequently affecting their antitumor activity [16]. These limitations have given rise to the need for alternative strategies, such as novel formulations and delivery systems, to improve the bioavailability of many phytocompounds, retain their parent structure, and increase their selective activity against cancer cells, providing maximum chemopreventive and chemotherapeutic effects [17,18].

In recent decades, nanoformulations have attracted the interest of many scientists and the pharmaceutical industry as a potential versatile, biocompatible, and biodegradable platform for drug delivery [19,20,21,22,23].

**Figure 1:**
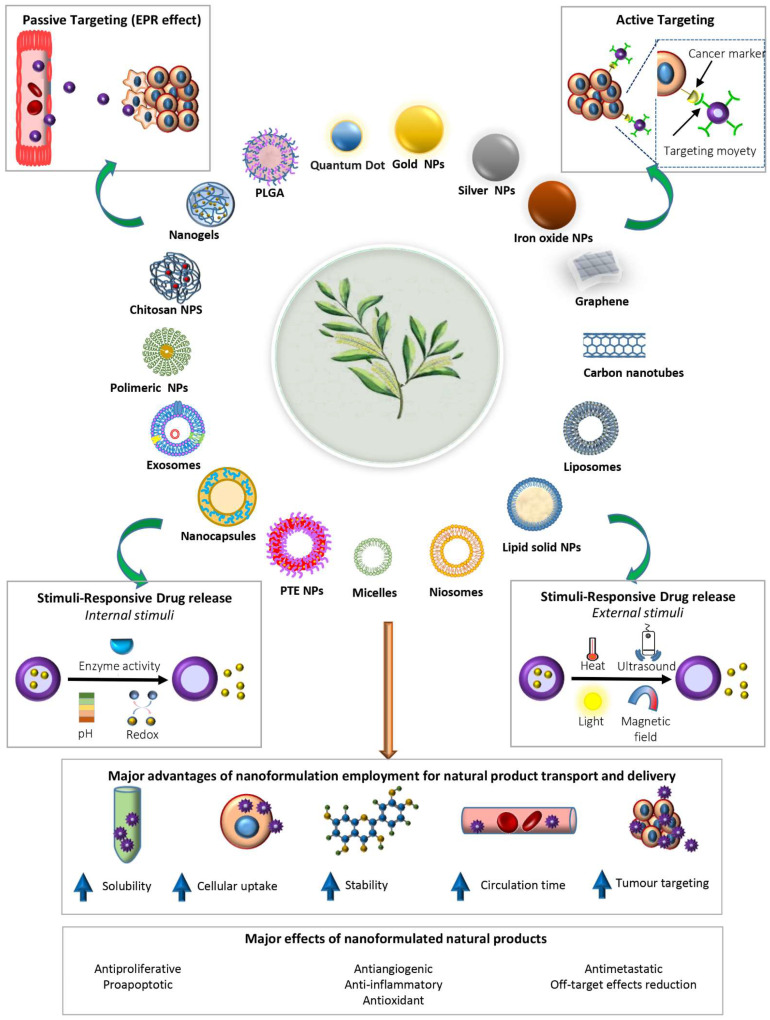
Pictorial representation of the main classes of nanoformulations for natural product transport and delivery. Schematic illustration of the passive, active targeting, and stimuli-responsive mechanisms of nano-based drug delivery systems, the stimuli-responsive drug release mechanisms, and the main advantages/effects of plants’ natural product nanoformulations’ employment in cancer therapy is reported.

Nanoformulations are composed of organic or inorganic nanostructures and nanomaterials such as polymer nanoparticles, lipid nanoparticles, micelles, and carbon nanotubes (CNTs) (Figure 1). This class of material has the characteristic that at least one of its dimensions lies between 1 to 100 nm. Owing to their nanoscale dimensions with a possible quantum limit, nanomaterials and nanostructures possess a high surface-to-volume ratio, rich surface/interface effects, and distinct physical and chemical properties compared with their bulk counterparts [24].

Nanostructure and nanomaterial features provide nanoformulations with additional properties; they ensure good penetration and protection of phytocompounds and/or xenobiotics from enzymatic degradation, facilitate their absorption, increase the efficiency of their loading, and extend their circulation time, thus improving their bioavailability and their enrichment in specific organs or tissues (Figure 1). For these reasons, nanostructures and nanomaterials play a crucial role in overcoming the challenge of bioavailability, aqueous solubility, the detrimental effects of environmental factors, such as fluctuation in pH, enzymatic attack, potential biochemical degradation, and low therapeutic absorption associated with phytocompounds as new chemical entities and drugs [19,20,21,22,23,25,26].

At present, the clinical application of most (about 97%) nanoformulations relies on tumor nanoparticle (NPs) accumulation through the passive trans-endothelial pathways from blood vessel to tumor tissue, such as via enhanced permeability and retention (EPR)-based tumor accumulation. However, EPR-based formulations have some disadvantages, such as nonspecific distribution, poor tumor accumulation, and the heterogeneity of tumors and patients. In addition, various stromal characteristics, such as extracellular matrix density, interstitial fluid pressure, variations in tumor blood flow and vascular permeability, growth-induced solid stress, and hypoxia, can have an additional impact on heterogeneity in EPR-based tumor targeting. As a result of all these variables, the EPR effect may not be applicable to all solid tumors [27,28]. In this context, researchers have concentrated their efforts on the assessment of new-generation nanoformulations with active targeting properties, such as ligand-based active tumor targeting and stimuli-responsive mechanisms (tumor microenvironment TME-responsive and exogenous stimuli-responsive drug delivery) [27,29] (Figure 1). The ligand-based targeting strategy is achieved through the decoration of the nanocarrier surfaces with ligands which selectively interact with receptors or antigens overexpressed onto the tumor cells. This strategy will improve the affinities of the nanocarriers for the surface of cancer cells, thus enhancing drug penetration. Moreover, the lack of such receptors or antigens in normal cells prevents nonspecific uptake by healthy cells and tissues. On the other hand, TME-responsive delivery systems exhibit an on-demand drug release profile upon response to stimulations from altered TME physiological features, which differ from healthy tissues. For instance, TME is characterized by acidic pH environment, hypoxic conditions, enzymatic variations, altered redox environment, elevated level of reactive oxygen, glutathione, adenosine triphosphate, and inflammatory factors.

In the case of exogenous stimuli-responsive systems, drug release can be induced and controlled by external factors such as temperature, light, magnetic fields, and ultrasound, which can be applied from outside the body directly to the tissue of interest, allowing for a potential circumvention of inter-tumor and inter-patient variability. Unfortunately, their application is limited in terms of tumor-targeting capability and induction of the proper release [29,30].

Finally, the physicochemical features of nanocarriers and their interaction with biological systems can be improved by modifying different characteristics such as their composition, shape, size, and surface charge [28].

In biomedical applications, multiple studies have demonstrated the synergistic effect of natural products in combination with conventional anticancer drugs [31]. The use of nanoformulations as delivery systems could further enhance this effect. Co-encapsulation of drugs with natural compounds in nanocarriers allows us to reduce the concentrations required for synergistic effects when compared with the combination of free substances. Furthermore, some co-encapsulated molecules, such as phenolic compounds, showed even higher biological activities compared with the pure free form [32]. The purpose of encapsulation can be different on the basis of cancer treatment strategies. A number of natural products were co-encapsulated to highlight synergistic effects with conventional antitumoral agents; moreover, some of them were used as chemosensitizers to overcome the multidrug resistance that represents a major challenge in cancer chemotherapy since it limits the effectiveness of many chemotherapeutic agents [33].

The purpose of our manuscript is to provide a comprehensive overview of the current research status and give an update on plant-derived natural products (phytocompounds), in particular secondary metabolites, as well as their main and recent nanoformulations. Indeed, the discovery of new compounds with potential anticancer activities and the application of nanotechnological devices for their transport and delivery are increasingly proving crucial means of overcoming the adverse effects of conventional chemotherapy such as systemic toxicity and multidrug resistance. This approach aims to achieve more-effective integrated therapeutic strategies.

## 2. Phytocompounds and Nanoformulations in Different Cancer Types

### 2.1. Breast Cancer

Breast cancer (BC) represents the most commonly diagnosed cancer in women in EU-27 countries in 2022 (13.8% of all cancers) and one of the most common cancer causes of death (7.5%) after lung and colon cancers [34] (https://joint-research-centre.ec.europa.eu/jrc-news-and-updates/cancer-cases-and-deaths-rise-eu-2023-10-02_en (accessed on 2 October 2023).

BC mortality rates in women are decreasing in most European countries thanks to the achievement of better-quality BC screening activities and treatment improvements. Despite this positive trend, BC incidence has been gradually increasing [35]. Therefore, numerous studies have now been carried out for years to search for additional and/or alternative BC targets and develop new treatment strategies.

BC treatment choice is strictly dependent on cancer stage (from stage 0, ductal carcinoma in situ, to stage IV, metastatic cancer) and molecular BC subtypes (Luminal A and B, triple-negative BC, HER2-positive, normal-like breast). BC patients are frequently subjected to combined treatments, including both standard modalities (surgery, radiation and chemotherapy, hormonal and targeted therapy), and complementary therapies (dietary planning and acupuncture), in order to achieve a safe, efficient, and personalized therapy [36]. Unfortunately, most commercially available drugs approved by the United States Food and Drug Administration (FDA) for BC chemotherapy are associated with reduced drug efficacy due to lack of selectivity, poor bioavailability, and induction of the multidrug resistance phenomenon leading to systemic toxicity, relapse, and disease recurrence [37]. Phytocompounds showed promising activities in preclinical BC models both in cancer prevention and therapy (see Table 1), reducing/overcoming the side effects [38].

Moreover, the combination strategy between standard chemotherapeutic drugs and phytocompounds has been found to be efficient for different subtypes, including the highly aggressive triple-negative BC (TNBC) [39].

#### 2.1.1. Phytocompounds

Among these bioactive compounds, cordycepin (3-deoxyadenosine, the main bioactive constituent of the fungus *Cordyceps militaris*) has been shown to induce both autophagic and apoptotic cell death, along with modulation of the epithelial–mesenchymal markers, suppression of Hedgehog and Notch signaling pathways in several BC cell lines as well as in mice-bearing xenografts [40]. Recently, this compound has been demonstrated to be effective against TNBC cell lines and in vivo tumor growth by inhibiting cell proliferation, EMT signaling pathways (TWIST1 and SLUG expression), and migration and invasion capabilities [41].

Curcumin (CUR) is a major natural low-molecular-weight polyphenol found in the rhizome of *Curcuma longa*. A number of studies have demonstrated its broad range of biological activities (antibacterial, antiviral, anticancer, anti-inflammatory, antioxidant), as well as its ability to induce both preventive and therapeutic effects in BC [42]. In the MDA-MB-231 cell line and mice bearing MDA-MB-231 xenografts, this polyphenol induced activation of the p53 signaling pathway, inhibition of angiogenesis, and suppression of the NF-κB signaling pathway. Moreover, several actions, such as modulation of cell cycle regulators, suppression of the PI3K/AKT/mTOR signaling pathway, induction of the mitochondrial apoptotic pathway, and suppression of the β-catenin signaling pathway, were detected in different BC cell lines upon CUR treatment [40]. CUR has been widely studied also in combination with conventional anticancer drugs (doxorubicin, paclitaxel (PTX), 5-fluorouracil (5-FU), cisplatin), showing itself to be both a chemopreventive and chemotherapy agent thanks to its multitargeting function on different regulatory molecules and key signaling pathways [43].

Epigallocatechin gallate (EGCG) represents the major catechin from the *Camellia sinensis* tea plant. Among the phytocompounds with anticancer activity, EGCG is one of the most studied for both in vitro and in vivo BC treatment [44]. EGCG anticancer activities include different cytotoxic mechanisms such as the induction of mitochondrial and death receptor apoptotic pathways, increase in pro-apoptotic and decrease in anti-apoptotic genes, induction of autophagy, and inhibition of angiogenesis [40]. Furthermore, thanks to the ability of EGCG in reducing the activity of receptors overexpressed in BC phenotypes, such as estrogen, progesterone, and HER2 receptors, it has also been employed in association with traditional chemotherapeutic agents, achieving a better treatment response due to a cancer cell sensitization. Combinations of EGCG with 5-aza 2′dC, oral cisplatin, or tamoxifen represent only a few examples of a synergistic response obtained in different BC cell lines and in tumor xenografts [44].

Diallyl disulfide (DADS, the major organosulfur compound from garlic *Allium sativum*) shows a good ability to inhibit proliferation both in BC cell lines and in animal models by inducing apoptosis and increasing the antioxidant cell defence [45]. Recently, the involvement of DADS and diallyl trisulfides (DATS) in regulating molecular mechanisms responsible for BC drug resistance has been widely demonstrated, mainly in TNBC [46].

Sulforaphane (SFN, an isothiocyanate organosulfur compound present in cruciferous vegetables such as *Brassica oleracea*, broccoli) exhibits both BC chemoprotective and anticancer effects through several mechanisms, such as induction of mitochondria-mediated apoptosis, cell cycle alteration, autophagy induction, and inhibition of angiogenesis and metastasis [47]. Recently, Cao et al. studied the role of SFN on BC metabolome and microbiome, showing its ability to regulate DNA methylation and gene expression [48]. Furthermore, the ability of SFN to target the BC cancer stem cell population (CSC) both in vitro [49] and in vivo [50] has been demonstrated for a few years through the inhibition of proliferation and mammosphere formation, as well as the decrease in CSC-related genes.

PTX is an antineoplastic drug approved by the FDA in 1994 for its employment in BC treatment [51]. Thanks to the active molecule isolated from the Pacific yew tree (*Taxus brevifolia*), PTX is able to bind to microtubules, interfering with their dynamics and polymerization, leading to a delay in mitosis progression, chromosomal segregation failure, and finally inducing mitotic block in G_2_/M phases and apoptosis [52]. Furthermore, several studies have demonstrated the additional effects underlying PTX anticancer activity, such as regulation of calcium signaling, which is responsible for cytochrome C release from mitochondria, and subsequent apoptosis, regulation of several miRNAs related to tumor progression, and modulation of immune response [53].

PTX, like the other taxane docetaxel and anthracyclines, is widely used both in neoadjuvant and adjuvant treatments in different BC subtypes, as well as in combination therapy with other chemotherapeutic drugs or anti-HER2 antibodies [54].

Unfortunately, PTX treatment is often associated with side effects and chemoresistance. Concerning the onset of toxicity effects, the main examples are represented by hypersensitivity (dyspnea, urticaria, bronchospasm, erythematous rash), cardiotoxicity (brady- and tachyarrhythmia, atrioventricular block, and cardiac ischemia) and neuropathies [55]. Concerning the induction of the MDR phenotype, several complex mechanisms have been demonstrated, underlying PTX resistance development; examples include, among others, ABC superfamily drug efflux protein over-expression, spindle assembly checkpoint protein and microtubule-associated protein altered expression, and changes in miRNA expression [53].

A number of in vitro and in vivo studies have shown that treatment strategies based on the combination of PTX and other phytocompounds seem to be effective in reducing BC drug resistance and side effects, as well as in achieving a synergistic tumor response [56]. Recently, the combination of PTX and oleuropein, a natural polyphenol found in olives and olive oils, has been evaluated in BC MCF-7 cell line. Authors found a synergistic anticancer effect after the combination treatment with the PTX concentration lower than IC_50_; in addition, cell antioxidant defense increased due to the reduction of several oxidative stress markers [57]. Furthermore, Zhang et al. showed that combination treatment of PTX with toosendanin, a plant-derived triterpenoid, synergistically reduced cell proliferation and induced apoptosis in MDA-MB-231 and BT-549 TNBC cell lines. In addition, it was able to inhibit both in vitro colony formation and migration ability and in vivo tumor growth more efficiently than PTX alone [58]. These results demonstrated that toosendanin strengthened the anti-tumor effect of PTX, suggesting that toosendanin/PTX combination could represent a promising alternative treatment strategy for patients with metastatic TNBC.

Cannabis and cannabinoids (CBs) from *Cannabis sativa*, a member of the Cannabaceae family, following their binding to specific receptors, exert their activity on the endocannabinoid system (ECS), responsible for correct tissue development and homeostasis maintenance in all stages of human body development (reproduction, embryogenesis, adult tissue activities, including the immune system) [59]. CBs have been used for years for pain relief, stimulation of appetite, and decreasing nausea in cancer patients. However, a number of in vitro and in vivo studies have shown that CBs show cytotoxic effects in different tumors, including BC, through several mechanisms (growth factor expression inhibition, cell cycle block/alterations, apoptosis induction, angiogenesis inhibition, invasion, and metastasis reduction) [60]. BC response to cannabinoid treatment was demonstrated to be strictly linked to BC subtypes depending on their different expression of the cannabinoid receptors. In particular, cell lines exhibiting a malignant phenotype and ER- status seemed to be more responsive to cannabinoid action [61]. Moreover, several pre-clinical studies in TNBC cell lines and mice xenograft models demonstrated that the main phytocannabinoids from *Cannabis sativa*, the ∆^9^-tetrahydrocannabinol (THC) and the non-psychoactive cannabidiol (CBD), induced antitumor effects (as cell proliferation inhibition, apoptosis/autophagy induction, migration/invasion reduction) through several mechanisms (receptor activation, increased ROS production, downregulation of NF-ĸB and MMPs, EGF/EGFR signaling inhibition, and downstream signaling pathway inhibition, such as AKT/mTOR and Raf-1/MEK/ERK) [62]. Interestingly, it has been demonstrated that CBs, mainly the synthetic ones, might induce cancer cell proliferation and survival when used at low doses, thus causing a biphasic behavior [60]. Among CBs, CBD has been also used in combination with chemotherapeutic drugs (PTX, mitoxantrone, temozolomide (TMZ), vinblastine, doxorubicin) in different tumors, including BC, achieving a reduction in the antineoplastic drug concentrations and a consequent decrease in drug side effects. Different studies showed the CBD/antitumor drug combination strengthened the drug antitumor efficacy both in BC cell lines and in vivo models; therefore, some clinical trials have been initiated to evaluate the effect of these combinations in BC and other tumors [63]. Other phytocompounds with demonstrated in vitro and pre-clinical activity, both in BC prevention and treatment, have been summarized by Svolacchia et al. [64].

**Table 1:** Phytocompounds and their main effects in breast cancer.

Phytocompounds	Source	Antitumor Mechanism	Refs.
Cordycepin (3-deoxyadenosine) 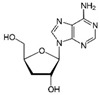	*Cordyceps militaris*	↑ autophagic and apoptotic cell death	[40]
		modulation of the epithelial–mesenchymal markers	
		↓ Hedgehog and Notch signaling pathways	
		↓ cell proliferation	[41]
		↓ EMT signaling pathways	
		↓ migration and invasion	
Curcumin 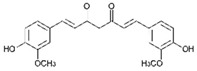	*Curcuma longa*	↑ p53 signaling pathway	[40]
		↓ angiogenesis	
		↓ NF-κB signaling pathway	
		modulation of cell cycle regulators	
		↓ PI3K/AKT/mTOR signaling pathway	
		↑ mitochondrial apoptotic pathway	
		↓ β-catenin signaling pathway	
Epigallocatechin gallate 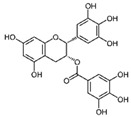	*Camellia sinensis*	↑ mitochondrial apoptotic pathways	[40]
		↑ pro-apoptotic genes	
		↓ anti-apoptotic genes	
		↑ autophagy	
		↓ angiogenesis	
Diallyl disulfide 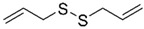	*Allium sativum*	↑ apoptosis	[45]
		↑ antioxidant cell defence	
		regulation of drug resistance	
Sulforaphane 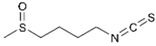	*Brassica oleracea*	Induction of mitochondria-mediated apoptosis	[47]
		induction of autophagy	
		↓ angiogenesis	
		↓ metastasis	
		Regulation of DNA methylation and gene expression	[48]
		Targeting of BC cancer stem cell population	[50]
Paclitaxel 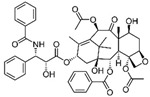	*Taxus brevifolia*	Interference with microtubule dynamics/polimerization	[52]
		Mitotic block/apoptosis miRNA regulation Immune response modulation Induction of drug resistance phenotype	[53]
Cannabinoids 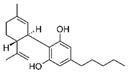	*Cannabis sativa*	↑ growth factor expression inhibition Cell cycle alterations	[60]
		Apoptosis induction ↓ Cell proliferation ↓ angiogenesis, invasion, metastasis ROS production ↓ NK-kB, MMPs ↓ AKT/mTOR, Raf-1/MEK/ERK pathways	[62]

#### 2.1.2. Nanoformulations

As reported in the Introduction, the employment of different drug delivery platforms allowed us to overcome, at least in part, those properties responsible for the low natural product treatment efficacy, mainly at the clinical level [65]. As the growth of BC subtypes is strictly dependent on the activity of several receptors (ErbB family, estrogen, folate, CD44, transferrin receptors), the receptor-mediated drug delivery represents the best therapeutic approach to achieve a selective result following the binding of a ligand–nanoparticle conjugate to the membrane receptors, the nanocarrier internalization through endocytosis, and the drug release via lysosomal degradation to the active sites of tumor cells [66].

Below, we report some recent examples of encapsulation and delivery strategies in BC treatment (see Table 2).

To overcome cordycepin low half-life due to the enzymatic degradation, its encapsulation in polymeric PLGA [poly (lactic-co-glycolic acid)] nanoparticles allowed for an increase of its half-life and an enhanced in vitro cytotoxic effect compared to the free compound. Cordycepin-loaded PLGA NPs did not induce hemolysis in an in vitro assay using rat RBCs, suggesting their possible use for intravenous administration [67]. Recently, Suksiriworapong et al. showed the improved cordycepin cellular uptake through endocytosis and the increased anticancer effect obtained against BC cell lines by lipid/polymer hybrid nanoparticles (LPNPs), obtained using both the polymeric material (PGA) and the lipid phosphatidylcholine. LPNPs were further decorated with hyaluronic acid (HA), a well-known CD44-targeting ligand frequently used to label the NPs’ surface for drug delivery to BC CD44-overexpressed cells [68].

A wide range of nanoformulations have been designed and evaluated for CUR encapsulation and delivery to BC. Huang et al. recently reviewed the currently used delivery systems based on the different BC targeting strategies. Systems such as liposomes, micelles, nanogels, polymeric NPs, exosomes, graphene oxide, and quantum dots, among others, are mostly employed in passive targeting (based on the enhanced permeability retention effect). Ligand or antibodies-conjugated delivery systems recognizing specific antigens/receptors on the BC surface allow for the active BC targeting, while chemical (pH) and physical (light, temperature, magnetism) stimuli represent intra- and extracellular conditions, respectively, for the physicochemical targeting strategy [69].

A number of nanoformulations have been designed to overcome EGCG poor adsorption, high degradation, and sensitivity to oxidation and hydrolysis [44]. Recently, the effect of EGCG encapsulated in lipid NPs functionalized with folic acid was studied in three BC cell lines (MCF-7, MDA-MB-231, and MCF-7TAM, resistant to tamoxifen) and normal MCF10A cells. In vitro studies regarding the intracellular LNP uptake, cell proliferation, and apoptosis induction showed that these NPs, loaded with EGCG, at very low concentrations, induced a significant cytotoxicity in the BC cell lines but not in MCF10A cells, suggesting their suitability also for in vivo applications [70]. Polymeric PLGA-NPs loaded with EGCG and decorated with the folate peptide (FP-EGCG-NPs) were evaluated both in vitro and in vivo, showing their higher efficacy as compared to free EGCG or unconjugated EGCG-NPs, confirming that nanocarrier conjugation with folate peptide represents a pivotal step towards achieving tissue selectivity [71].

Despite the strong anticancer activity of DADS and DATS, these compounds show severe side effects, such as hemolytic effects, and possible interaction with serum or digestive system components that strongly limit their in vivo application. Several nanoformulations, reviewed by Pandey et al. [72], have been designed to overcome these problems. (HA)-based nanocapsules were designed as efficient DADS and DATS delivery systems in a mouse mammary gland cancer cell line that mimics human BC stage IV (4T1), allowing for natural compound enhanced stability and avoiding erythrocyte lysis [73]. Solid lipid NP formulation was employed to deliver DADS to the MDA-MB-231 cell line selectively, selected as a TNBC cell model [74]. Another study employing solid lipid NPs carrying DATS took advantage of folate receptor overexpression on TNBC cells to selectively target this BC subtype. In vitro evaluation of DATS-SLNPs functionalized with folic acid revealed that a higher cytotoxic effect, a delayed cell migration, an improved DATS internalization, and an increased apoptotic response could be detected in MCF-7 and MDA-MB-231 cells [75]. Gunasekaran et al. reported the anticancer activity of nanoliposomes containing cisplatin and DADS against the MDA-MB-231 cell line. An increased cytotoxic effect and metastasis inhibition was induced by dual drug-loaded nanoliposomes (lipo-CDDP/DADS), even with low drug concentration, as compared with free drugs and lipo-CDDP or lipo-DADS [76].

The low bioavailability and solubility of SFN has stimulated further studies focused on improving their intracellular absorption and therapeutic efficacy against BC, alone or in combination with other drugs/compounds. A co-delivery approach involving SFN and CUR was designed, employing PEGylated gold-coated Fe_3_O_4_ magnetic nanoparticles (PEGylated Fe_3_O_4_@Au NPs) as a delivery system in the MCF-7 cell line [77]; furthermore, histopathology analysis of different tissues showed a lack of toxicity after intravenous injection to female BALB/c mice. In another study, SFN encapsulation in Poly (caprolactone)- poly (ethylene glycol)- Poly (caprolactone) (PCL–PEG–PCL) copolymeric-based micelles induced the following effects: (i) higher in vitro cytotoxic effect and apoptosis when compared to free SF; (ii) greater in vivo tumor growth inhibition in a mice 4T1 BC model compared with free SF [78]. A number of studies have assessed the combined delivery of SFN with other anticancer drugs, such as doxorubicin, taxanes, and tamoxifen, by employing several nanoformulations aimed at reducing the drug concentration and their systemic toxicity, increasing the final drug therapeutic efficacy [79].

The poor solubility and the side effects associated with conventional PTX treatment have strongly driven the search for drug combinations and/or PTX transport and release to tumor sites. The development of several drug delivery systems (DDS) for PTX has partly allowed the reducing of its negative effects, improving its therapeutic potential.

Albumin-bound PTX (Nab-P, Abraxane^®^, American BioScience, Inc., Santa Monica, CA, USA) was the first PTX-based nanoformulation approved by the FDA for the treatment of advanced breast cancer. Solvent-free Nab-P is characterized by selective albumin delivery to tumors through transcytosis (receptor-mediated transport) [80]. A number of studies have largely demonstrated that Abraxane^®^ exhibits increased efficacy and tolerance in patients with stages of BC as compared to conventional PTX [81].

Since Abraxane^®^’s marketing, numerous other DDS have been designed and developed for PTX employment, alone or in combination with other phytocompounds. Among these nanoformulations, functionalized PTX-encapsulated PEGylated PLGA NPs, PTX-loaded folate-coated pH-sensitive liposomes, PTX-encapsulated tannic acid NPs, folate-conjugated curcumin and PTX-loaded lipid NPs, and PTX-naringin co-encapsulated in mixed polymeric micelles are just a few examples [53].

In a recent work from Nicoud et al., a novel nanomicellar polymeric formulation containing the biocompatible copolymer Soluplus^®^ (BASF, Buenos Aires, Argentina), surface-coated with glucose and co-loaded with histamine and/or PTX, was designed and tested for its antitumor efficacy both in vitro and in vivo. Cytotoxicity assays performed in human MDA-MB-231 and murine 4T1 cell lines and in vivo treatment of a TNBC model showed that this nanoformulation was effective in inducing apoptotic cell death and reducing tumor volume and neovascularization, suggesting that it could represent a new DDS strategy for BC chemotherapy [82]. Velhal et al. recently reviewed the employment of different cyclodextrin-conjugated PTX nanoparticles for the treatment of different tumors, including BC. In vitro and in vivo studies evaluated their efficacy, confirming that a cyclodextrin-based delivery system for PTX can be considered promising, although no conclusive clinical trial results have yet been obtained [83].

Different delivery systems have been employed in order to improve the efficacy of cannabinoids by overcoming the problems associated with their use, such as low solubility/bioavailability, high instability, resistance mechanism induction, and rapid metabolism. Several cannabis-based nanoformulations are currently under study both for preclinical assessment and clinical development, including lipid-based carriers (liposomes, micelles, NLC, and lipid nanocapsules), self-emulsifying DDSs, and polymeric carriers (PLGA) [84].

Fraguas-Sánchez et al. evaluated the anticancer activity of CBD loaded into PLGA microparticles and administered to BC cell lines (MCF-7 and MDA-MB-231) in combination with either PTX or doxorubicin. This nanoformulation was shown to be highly effective against cancer cell proliferation; in addition, its activity was prolonged over time as compared to CBD administered in solution in combination with the antitumoral drugs [85]. These promising results were also obtained in MDA-MB-231-derived tumors (chick chorioallantoic membrane model), confirming the status of PLGA microparticles as an innovative tool for CBD/anticancer drug prolonged release in both ER^+^ and TNBC.

In a recent study, CBD was combined with 20(S)-protopanaxadiol (derived from the Chinese herbal medicine Ginseng) and loaded into liposomes, previously modified to expose glucose residue on the surface in order to target the GLUT1 receptor, frequently over-expressed on tumor cells [86]. In vivo studies on murine breast tumor (4T1 cells)-bearing BALB/c mice showed that this nanoformulation exerted increased anticancer activity, as compared with the two compounds alone, by inhibiting tumor growth, and it was also characterized by a good tolerance. Therefore, this combined therapy could represent a new treatment strategy for aggressive and metastatic BC.

**Table 2:** Phytocompound-based nanoformulations (NF) and their main advantages in breast cancer.

Phytocompounds	NF	Advantages	Refs.
Cordycepin(3-deoxyadenosine)	PLGA NPs	Increased half-life Enhanced cytotoxic effects	[67]
	Lipid–polymer hybrid NPs	Higher intracellular uptake Improved delivery	[68]
Curcumin	Liposomes Micelles Nanogels Polymeric Nanoparticles Exosomes Graphene oxide Quantum dots	Increased half-life/stability/water Solubility Enhanced in vivo distribution and Tumor accumulation Improved tumor targeting	[69]
Epigallocatechin Gallate	Folic acid-functionalized Lipid NPs	Enhanced cytotoxicity Increased circulation time Improved tumor targeting	[70]
	Folate peptide-decorated PLGA NPs	Improved cellular internalization Increased half-life Enhanced cytotoxicity Improved in vivo selectivity and efficacy	[71]
Diallyl disulfide	HA-based NPs	Improved water solubility and stability Reduced oxidation and blood components interaction	[73]
	SLN NPs	Increased bioavailability Improved selective delivery Enhanced apoptosis	[74]
	Folic acid-functionalized SLN NPs	Increased bioavailability Improved cell internalization Increased apoptosis Delayed migration	[75]
	Nanoliposomes (DADS + cisplatin)	Increased stability Enhanced cytotoxicity Reduced metastatic activity	[76]
SFN	PEGylated Fe_3_O_4_@Au NPs (SFN + CUR)	Increased water solubility Enhanced synergistic effect Increased cytotoxicity	[77]
	PCL-PEG-PCL micelle	Increased cytotoxicity Prolonged circulation time Improved in vivo tumor growth inhibition	[78]
Paclitaxel	Albumin bound (Abraxane)	Enhanced endothelial cell binding and transcytosis Improved intratumor accumulation Increased in vivo anticancer efficacy	[80,81]
	PEG-PLGA NPs pH sensitive liposomes Lipid NPs Polymeric micelles	Increased biocompatibility Enhanced cellular uptake Reduced side effects MDR overcoming multiple drug synergism	[53]
	Nanomicellar polymeric formulation	Reduced cell proliferation Enhanced apoptosis Inhibited cell migration Reduced in vivo tumor growth/vascularization	[82]
	Cyclodextrin NPs	Improved water solubility and drug stability Reduced off-target effects enhanced cytotoxicity	[83]
Cannabinoids	Lipid-based carriers, self-emulsifying DDSs	Increased half-life Improved stability Controlled release at target sites Reduced systemic toxicity	[84]
	Polymeric carriers PLGA Microparticles (CBD)	Reduced cell proliferation Prolonged drug activity Enhanced antitumor efficacy	[85]
	Liposomes [CBD+20(S)-protopanaxadiol]	Increased anticancer activity, multiple drug synergism, and cancer cachexia modulation	[86]

### 2.2. Colorectal Cancer

Colorectal cancer (CRC) is the third most common cancer in the world, especially in people over 50 years of age, and represents the second leading cause of cancer death [1] (WHO—1 February 2024).

High intake of processed meat, reduced consumption of fruit and vegetables, sedentary lifestyle, obesity, smoking, and excessive alcohol consumption are considered the main risk factors that contribute to the onset of CRC. Moreover, inflammatory bowel diseases can disrupt the mucosal barrier and induce the release of pro-inflammatory and carcinogenic mediators, increasing the possibility of developing CRC [87].

CRC is often diagnosed at an advanced stage, when treatment options are rather limited; patients in whom CRC was diagnosed at an early stage have a greater chance of survival than those in whom it was diagnosed later [88]. Based on tumor progression, it is possible to choose the most suitable therapy: laparoscopic surgery for early-stage CRC, open surgery for metastases. Advanced CRC is treated with chemotherapy drugs, such as 5-FU, oxaliplatin, irinotecan, and monoclonal antibodies such as cetuximab or bevacizumab (https://www.cancer.gov/about-cancer/treatment/drugs/colorectal (accessed on 1 February 2024)). However, the effectiveness of therapy is very often limited by the onset of drug resistance due to epigenetic factors, changes in drug metabolism, increases in drug efflux mediated by ATP-binding cassette transporters, increased DNA repair, and autophagy induction [89]. To overcome multidrug resistance, new alternative approaches, such as phytocompounds and nanoformulations, are being evaluated (see Table 3) [90,91].

In vitro and in vivo studies demonstrated that phytocompounds can induce apopototic cell death and can affect cell proliferation, migration/invasion, angiogenesis, and metastasis, targeting multiple signaling pathways and regulating the expression and function of miRNAs in CRC [92]. Furthermore, the high fiber content in fruit and vegetables, which contributed to reducing the risk of CRC, justified the importance of the in-depth study of natural substances and food supplements as chemopreventive agents to treat CRC [90].

#### 2.2.1. Phytocompounds

Our previous works demonstrated that the extract of *Prunus pinose* drupes (typical plant of Molise, IT) was effective on 2D, 3D, and in vivo CRC models. Analysis of the chemical composition of the compound (Trigno M^®^, Biogroup, Isernia, Molise, Italy) showed that the main active components were phenolic acids, flavonoids, and anthocyanins, known for their antioxidant and antiproliferative activities [93]. Monotherapy with this natural compound (Trigno M^®^) reduced HCT116 cell viability, induced apoptosis on 2D and 3D models, and reduced tumor growth in immunodeficient xenografts mice carrying colon rectal cancer [94]. When Trigno M^®^ was administered in combination with 5-FU, we confirmed the induction of apoptosis on 2D and 3D models and we demonstrated the inhibition of autophagy-mediated resistance [95].

*Rosmarinus officinalis* L. extract showed an antiproliferative effect on colon cancer cells, upregulating the glucosaminyltransferase 3 (GCNT3), an essential molecule for CRC progression, and downregulating its potential epigenetic modulator miR-15b [96,97].

Resveratrol (Resv, trans 3,5,4′-trihydroxy-stilbene), a polyphenol of the stilbenes group, is a non-flavonoid compound found in many plant species, such as grapes, red fruits, and peanuts. It is produced in plants in response to viral and fungal infection, UV radiation and mechanical injury [98]. Resv was an effective chemosensitizer agent for CRC [99]; in addition, it chemosensitized 5-FU-resistant HCT116 cells, inducing caspase-3-dependent apoptosis; it strongly suppressed TNF-β-induced activation of tumor-promoting factors (as NF-κB, MMP-9, and CXCR4) and epithelial-to-mesenchymal transition factors (increased vimentin and slug, decreased E-cadherin). Moreover, Resv suppressed the formation of cancer stem cells, decreasing CD133, CD44, and ALDH1 expression. A more recent work demonstrated that its chemosensitizing effect was also due to modulation of the β1-integrin/HIF-1α axis that was highly pronounced in CRC [100]. The antitumoral and chemopreventive effect on CRC was also due to activation of autophagy, as demonstrated by the study in HT-29 and COLO 201 human colon cancer cells. Transmission electron microscopy observations and immunoblotting tests demonstrated that Resv induced autophagy, which then induced apoptosis mediated by ROS production [101].

Quercetin (QUE), contained in many fruits and vegetables, suppressed the proliferation of CRC cells and induced apoptosis, especially in cells with activated mutation in the KRAS gene [102]. This mutation, found in 40% of CRC, was associated with resistance to conventional and targeted chemotherapy [103]. QUE antitumor effect was ascribed to AKT inhibition and JNK signaling activation [102].

In vitro studies demonstrated the double role of SFN against CRC: on the one hand, it reduced SW480, DLD1, and HCT116 cell growth by modulating the Wnt/β-catenin pathway with an antitumoral effect [104]; on the other hand, as a phytoantioxidant compound, it protected CRC cells from oxidative stress, activating the Nrf2-mediated cytoprotective mechanism [105].

The molecular pathways involved in CUR anticancer and chemopreventive properties against CRC have been widely clarified, including Wnt/β-catenin, JAK, STAT, MAPK, and NF-kB pathways [106,107]. When used in combination with traditional chemotherapeutics, as oxaliplatin and 5-FU, CUR was able to overcome chemoresistance both in in vitro and in vivo CRC models. For example, oxaliplatin plus CUR reversed the resistance of the CRC cell lines modulating the chemokines/NF-kB signaling pathway [108]. Alternatively, CUR reversed the 5-FU resistance of HCT116 cells inhibiting epithelial–mesenchymal transition progress, acting on the WNT signaling pathway [109].

CBs represent another example of natural compounds with promising effects in CRC [110]. A recent study analyzed the endocannabinoid system, and cannabinoid receptor 2 (CB2 in mice and CNR2 in humans) in particular [111]. The results demonstrated that the activation of endogenous CB2 with cannabinoids modulated the immune response and inhibited colon tumorigenesis. Consequently, this study identified CB2 as a potential target for CRC personalized therapy. An in vitro and in vivo study demonstrated that CBD acted on CRC proliferation, invasion, and metastases. CBD inhibited EMT process acting on the Wnt/β-catenin signaling pathway; in particular, it downregulated APC and CK1 expression and upregulated Axin-1 [112]. Another study on the glycosidic derivative of Δ^9^-tetrahydrocannabinol (THC-9-OG), employing both in vitro and in vivo models, also confirmed the action on EMT when cannabinoids were administered in combination with 5-FU. Moreover, the combination of THC-9-OG plus 5-FU induced ROS production, causing ATM activation and vimentin downregulation, activating autophagic cell death [113]. CBD was also combined with photodynamic therapy for CRC treatment. The combination increased oxygen reactive species, inducing apoptotic cell death. Moreover, it stimulated an immune response that interfered with specific mechanisms regulating CRC tumorigenesis, drug resistance, and metastasis [114].

It is known that betulinic acid (BA) behaves as a chemosensitizer, in combination with 5-FU, irinotecan, and oxaliplatin, on CRC drug resistant cells, inducing mitochondrial-dependent apoptosis [115]. Subsequent studies have shown that BA also displayed a chemopreventive effect on a CRC animal model. COX-2 level and PCNA expression decreased in animals treated with BA plus 1,2dimethylhydrazine (DMH) (carcinogen compound) compared to those treated with DMH alone. So, the chemopreventive effect was ascribed to antiproliferative and anti-inflammatory actions [116]. A recent study investigated via transcriptome analysis the mechanism by which BA induced an antiproliferative effect on CRC. The upregulation of metallothionein 1G (MT1G) induced cell cycle alteration and inhibited cell proliferation [117].

**Table 3:** Phytocompounds and their main effects in colorectal cancer.

Phytocompounds	Source	Antitumor Mechanism	Refs.
Trigno M^®^	*Prunus spinosa*	Antioxidant and antiproliferative activities	[93]
		↓ cell viability	[94]
		↓ tumor growth	
		↑ apoptosis	
Rosemary extract	*Rosmarinus officinalis* L.	↓ cell proliferation	[95,97]
		↑ glucosaminyltransferase 3	
		↓ miR-15b	
		Chemosensitization	[99]
Resveratrol 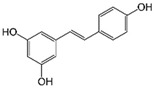	Grapes, blueberries, raspberries, mulberries, peanuts	↑ caspase-3-dependent apoptosis	[99,100]
		↓ tumor-promoting factors (as NF-κB, MMP-9, CXCR4)	
		↓ epithelial-to-mesenchymal transition factors	
		↓ cancer stem cells	
		↓ CD133, CD44, and ALDH1 expression	
		Modulation of β1-integrin/HIF-1α	
		↑ autophagy	[101]
		↑ ROS production	
Quercetin 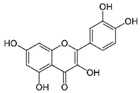	Citrus fruits, apples, onions, parsley, sage, tea, red wine	↓ cell proliferation	[102]
		↑ apoptosis	
		↓ AKT signaling	
		↑ JNK signaling	
Sulforaphane 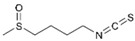	*Brassica oleracea*	↓ cell growth by modulating Wnt/beta-catenin pathway	[104]
		↑ Nrf2-mediated cytoprotective mechanism	[105]
Curcumin 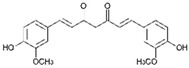	*Curcuma longa*	Regulation of Wnt/β-catenin, JAK, STAT, MAPK, and NF-kB pathways	[106,107]
		Reversion of 5-FU resistance	[109]
		↓ epithelial–mesenchymal transition	
Cannabinoids 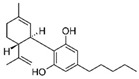	*Cannabis sativa*	Antiproliferative activities and chemosensitization ↓ epithelial-to-mesenchymal transition factors ↓ cell growth by modulating Wnt/beta-catenin pathway	[112]
		↑ autophagic cell death	[113]
Betulinic acid 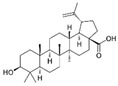	Birch, eucalyptus, plane trees	Chemoprevention and chemosensitization ↑ mitochondrial-dependent apoptosis	[115]
		↓ COX2 level and PCNA expression ↓ metallothionein	[117]

#### 2.2.2. Nanoformulations

Due to poor water solubility, low chemical stability, and short biological half-life, the use of phytocompounds in CRC clinical therapy is limited. The encapsulation in nanocarriers allowed us to overcome these limitations and improve their anticancer efficacy (see Table 4) [118].

To this end, Resv-loaded mesoporous silica NPs were developed. Moreover, their surface conjugation with HA enhanced Resv anticancer efficacy due to increased specific binding to tumor receptors and improved cellular internalization [119]. Resv was also encapsulated in tecnetium-99m-labeled gold nanoparticles. In vitro and in vivo study showed a higher Resv gold nanoparticles cellular uptake as compared to the compound alone [120]. Finally, some evidence indicates that Resv can be co-encapsulated with cyclodextrin in liposomes, resulting in enhanced toxicity as compared to free Resv in CRC cells [121].

In addition to Resv, other polyphenols with promising activities against CRC have been encapsulated in nanocarriers as micelles, nanogels, liposomes, nanoemulsions, AuNPs, mesoporous silica, and metal–organic nanoparticles [122].

To improve QUE release rate and anticancer effect, it was loaded into the oil phase of the nanoemulsion using D-tocopheryl polyethylene glycol succinate as a surfactant [123].

Nanoemulsion-loaded QUE proved to be more effective in inhibiting CRC cell viability than the drug alone. Furthermore, in vivo studies showed that this nanoformulation was able to inhibit inflammation of the intestinal mucosa caused by various chemotherapeutics, such as 5-FU, by hampering the formation of reactive oxygen species and decreasing NF-kB and HIF-1alpha expression [124].

To optimize its antitumoral effect, SFN-loaded gold nanoparticles were developed. In vitro data showed that SFN-loaded gold nanoparticles were more effective than SFN alone. In vivo study demonstrated an increased retention of SFN-loaded gold nanoparticles in tumor site even after 24 h of treatment and a noticeable tumor reduction with SFN-loaded gold nanoparticles compared to SFN alone [125].

Although many ongoing clinical studies testified that CUR could be particularly successful in the treatment of CRC, the limits linked to its poor solubility in water encouraged new studies to search for nanoformulations to improve CUR transport and delivery [126]. For example, CUR were loaded in niosomal nanoparticles, achieving an encapsulation efficacy of approximately 80%. Niosomal nanoparticles showed no toxicity against SW480 cells, whereas CUR-loaded niosomal NPs were found to be effective against CRC cells, increasing pro-apoptotic protein expression [127]. Another recent study showed that encapsulation of CUR in mannosylated chitosan NPs enhanced CUR anticancer effect against HCT116 and SW480 human cell lines through activation of caspase signaling and apoptotis induction [128]. In addition, this CUR nanoformulation showed no effect against non-malignant cell lines, suggesting its possible safe employment for clinical applications.

Δ^9^-THC was encapsulated in PLGA NPs (THC-PLGA NPs) conjugated, or not, with transferrin. Both nanoparticles were internalized via clathrin-mediated mechanisms. The transferrin-modified form induced greater toxicity than the unmodified form. The addition of transferrin favored a prolonged action on the surface of the target cell [129].

To deliver BA, several kinds of nanovectors (polymeric and magnetic NPs, liposomes, nanoemulsions, carbon nanotube) were used [130]. A promising BA analogue was encapsulated in polymeric nanocarrier, and its therapeutic efficacy was evaluated on a CRC in vitro and in vivo model. The results demonstrated that the induction of apoptosis was greater in the encapsulated form than in the free form [131]. Wang et al. encapsulated BA in pH-sensitive liposomes, demonstrating that this formulation inhibited tumor growth and increased immunity levels of tumor-bearing mice [132]. Finally, BA was incapsulated in the mitochondrial-targeted nanocomplex, i.e., modified gold NPs, which were able to inhibit tumor growth more efficiently than free BA [133].

**Table 4:** Phytocompound-based nanoformulations (NF) and their main advantages in colorectal cancer.

Phytocompounds	NF	Advantages	Refs.
Resveratrol	Mesoporous silica NPs	Increased solubility Improved release Reduction of NF-kB expression Increased apoptosis	[119]
	Tecnetium-99m labeled gold nanoparticle	Increased uptake	[120]
	Liposomes	Improved release	[121]
		Enhanced cytotoxicity	
Quercetin	Nanoemulsions	Inhibition of inflammation Decreased NF-kB and HIF-1α expression	[124]
Sulforaphane	Gold NPs	Noticeable tumor reduction	[125]
Curcumin	Niosomal NPs	Increased pro-apoptotic protein expression	[127]
	Mannosilated chitosan NPs	Enhanced caspase-activation and apoptosis induction	[128]
Cannabinoid	PLGA NPs	Increased release	[129]
Betulinic acid	pH-sensitive liposomes	Enhanced tumor growth inhibition	[132]
		Increased immunity stimulation	
	Gold NPs	Significant inhibition of cancer cell growth	[133]

### 2.3. Lymphomas

Lymphoma, which accounts for approximately 5% of all malignancies, includes a heterogeneous group of neoplasms arising from the clonal proliferation of lymphocytes. The exact cause of lymphoma is often elusive, but factors including immune system dysregulation, infections, and autoimmune diseases are recognized to increase the risk of lymphoma development significantly.

The WHO classification system distinguishes between lymphoid neoplasms derived from precursor lymphoid cells and those derived from mature lymphoid cells, considering neoplasms of B or T cell origin [134]. The two main categories of mature lymphoid neoplasms are Hodgkin’s lymphoma (HL), 10%, and non-Hodgkin’s lymphoma (NHL), 90% [135]. HL is characterized by the presence of Reed–Sternberg cells, mainly in B-cell lymphoma, and is divided into two distinct categories: classical and nodular lymphocyte-predominant Hodgkin lymphoma (NLP-HL).

Even though targeted agents have shown remarkable success in treating lymphoma patients, their side effects are still an obstacle to safe therapy [136,137].

#### 2.3.1. Phytocompounds

Natural products derived from plants, fungi, and marine organisms have attracted considerable attention for their potential to treat various diseases, including lymphoma (see Table 5) [138]. Among the phytocompounds, polyphenols represent the most studied for their multi-target antitumor activities regulating the angiogenesis, inflammation, and apoptosis of CSCs in vitro [139]. Moreover, polyphenols may enhance immune responses through the modulation of T lymphocytes.

Recent studies on natural polyphenols have shown that they can be an adjuvant to conventional therapy to limit the CSCs drug-resistance phenomenon. In this context, CUR suppresses cell proliferation by inhibiting STAT-3 and NF-kB, inducing apoptosis and cell cycle arrest and inhibiting angiogenesis [140,141]. Furthermore, studies reported that CUR treatment increased caspase-3 and caspase-9 levels in CH12F3 cells, effectively reducing cell-proliferation activity [142]. Similarly, CUR has been found to inhibit cell viability, promote cell apoptosis, and arrest the cell cycle in the G2 phase of human DLBCL cells both in vitro and in vivo by upregulating the expression of PPARγ and deactivating the Akt/mTOR pathway [143]. In vivo anticancer effects of CUR on human Burkitt’s lymphoma (Raji cells) have been reported in a xenograft mouse model, resulting in the downregulation of c-Myc oncogene and the upregulation of apoptotic proteins [144].

Different studies have shown the Resv anti-inflammatory, antioxidant, and cell cycle arresting properties in lymphomas. Resv suppresses tumor cell viability by inhibiting the ROS-dependent PI3K/AKT signaling pathway [145]. Frazzi et al. reported that Resv treatment induced cell cycle arrest and apoptosis in the HL-derived L-428 cell line, increasing the expression of p53 and p53 target genes, including Bax and caspase-3 [146]. Resv induced apoptosis through ROS generation in DLBCL cells, dephosphorylating the survival mediator Akt, the transcription factor FOXO1, GSK3, and the apoptotic mediator Bad [147]. Another study reported Resv anti-cancer effects on the anaplastic large-cell lymphoma (ALCL) cell line SR-786 through the increase in Fas/CD95 expression in a dose-dependent manner [148]. In addition, Resv induced cell cycle arrest and apoptosis in malignant NK cells by suppressing the JAK2/STAT3 pathway [149]. Kong et al. reported that pterostilbene, a natural analogue of resveratrol, induced apoptosis in SUDHL-4 and NU-DUL-1 cell lines by activating the Bax/Bcl2 pathway. Pterostilbene significantly induced cell cycle arrest at the G1 phase by inhibiting cyclin-dependent kinase-2 (Cdk-2) and enhancing the effect of checkpoint dependent kinase-2 (Chk-2). In addition, the intravenous administration of pterostilbene inhibited tumor growth in the nude mouse xenograft model [150].

Granato et al. suggested that QUE inhibited the PI3K/AKT/mTOR and STAT3 pathways in primary effusion lymphoma (PEL), an aggressive B-cell lymphoma, reducing the expression of pro-survival cellular proteins such as c-FLIP, cyclin D1, and c-Myc, and the release of the cytokines IL-6 and IL-10, leading to PEL cell death [151]. In addition, this flavonoid induced hyperactivation of PI3K signaling in ascites cells from mice with Dalton’s lymphoma, activating AKT1 and inactivating p53. Glycolytic metabolism was also downregulated by QUE [152]. Furthermore, QUE increased in vitro apoptotic human T lymphoblast MOLT-4 (acute lymphoblastic leukemia) and human B lymphoblast Raji (Burkitt’s lymphoma). Li et al. reported that QUE, in combination with rituximab, increased apoptosis and inhibited cell growth in DLBCL cell lines, potentiating the anti-tumor effect of rituximab through STAT3 pathway inhibition and Mcl-1 and Bcl-xl expression decreases [153].

**Table 5:** Phytocompounds and their main effects in lymphoma.

Phytocompounds	Source	Antitumor Mechanism	Refs.
Curcumin 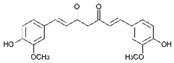	*Curcuma longa*	↓ cell proliferation	[140,141,143]
		↓ STAT-3 and NF-kB	
		↑ apoptosis	
		cell cycle arrest	
		↑ PPARγ expression	
		↓ Akt/mTOR pathway	
		↓ angiogenesis	
		↑ caspase-3 and caspase-9	[142]
Resveratrol 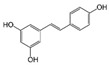	Grapes, blueberries, raspberries, mulberries, peanuts	Cycle arresting ↓ cell viability ↓ ROS-dependent PI3k/Akt signaling ↑ apoptosis	[145,146,149]
		↑ p53 and p53 target genes ↑ Bax and caspase-3	[146]
		↑ ROS production ↓ Akt, FOXO1, GSK3 and Bad phosforilation	[147]
		↑ Fas/CD95 expression	[148]
		↓ JAK2/STAT3 pathway	[149]
Quercetin 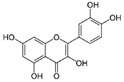	Citrus fruits, apples, onions, parsley, sage, tea, red wine	↓ PI3K/AKT/mTOR and STAT3 pathways	[151]
		↑ cell death	
		↓ c-FLIP, cyclin D1 and c-Myc expression	
		↓ release of IL-6 and IL-10	
		↑ PI3K signaling	[152]
		↑ AKT1 activation	
		↓ P53 activation	
		↓ glycolytic metabolism	
Cannabinoid 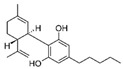	*Cannabis sativa*	↓ cell proliferation	[154]
		↑ apoptosis	
		↑ NADH	
		↑ ROS production	
		↓ GSH	
		↑ caspase-3 ↑ apoptosis	[155]

Furthermore, some studies have also provided evidence that CBs, such as CBD and Δ^9^-tetrahydrocannabinol, reduced the growth and survival of lymphomas by disrupting various cellular signaling pathways involving type 1 (CB1) and type 2 (CB2) cannabinoid receptors [154]. Gustafsson et al. have proved that R(+)-methanandamide (R(+)-MA) induced apoptosis in MCL and CLL cell lines, which express higher levels of CB1 and CB2 cannabinoid receptors and significant inhibition of in vivo tumor growth [155]. Moreover, in vivo studies showed that treatment of EL-4 murine lymphoma-bearing mice with THC reduced cell viability and tumor burden and increased the survival of tumor-bearing mice [156]. Strong et al. have proved that CBD in combination with well-known chemotherapeutics, such as ibrutinib, carfilzomib, and Tumorex, displayed synergistic potential in the treatment of DLBCL and MCL cell lines [157]. However, as for other malignancies, despite their proven efficacy, the potential health benefits of phytocompounds are limited and the optimization of delivery systems to target lymphoma cells could be an approach with potential clinical significance.

#### 2.3.2. Nanoformulations

As concerns nanoformulations (see Table 6), an in vivo study reported that formulation of CUR in solid lipid nanoparticles (SLN) or d-α-tocopheryl polyethylene glycol 1000 succinate (TPGS) nanoparticles, administered in the nude mouse xenograft model, reduced tumor growth of L-540 human Hodgkin’s lymphoma cancer cells. SLN-CUR significantly reduced the in vitro expression of Mcl-1 and XIAP and the cytokines IL-6 and TNF-α involved in HL lymphoma proliferation [158]. Guo et al. reported that the mPEG-b-P (Glu-co-Phe) nanoparticles, co-loaded with doxorubicin and CUR, showed a high anti-lymphoma effect on BJAB and Raji cells by enhancing the apoptotic pathway and reducing the invasive ability of B-cell lymphoma in the nude mouse xenograft model [159].

Co-administration of CUR with imatinib in nanolipid carriers (NLCs) reduced the amount of imatinib needed to treat malignant NHL cells. In addition, the use of rituximab as a targeting agent for CUR/imatinib/NLCs delivery to malignant NHL cells induced significant cytotoxic effect in Ramos B cells (CD20 receptor positive) but not in Jurkat T cells (CD20 receptor negative) due to the increased cellular uptake [160].

Radeva et al. demonstrated the effectiveness of double-loaded doxorubicin/Resv polymeric micelles in the treatment of L5178 lymphoma cells resulting in higher cytotoxicity and lower cardiotoxicity in lymphoma cells [161]. An in vitro study demonstrated that pretreatment of lymphoma cell lines with Resv could downregulate the expression of anti-apoptotic proteins Bcl-xl, sensitizing cells to paclitaxel-mediated apoptosis. Furthermore, on the other hand, Resv increased the expression of pro-apoptotic proteins Apaf-1 and Bax [162].

Yu et al. showed the antitumor activity of pterostilbene against Mantle cell lymphoma (MCL) through dose-dependent cell proliferation inhibition. In MCL cell lines and mouse models, they suggested that the pterostilbene and bortezomib combination leads to synergistic cytotoxicity [163]. Zhu et al. found that QUE and vincristine (VCR) loaded on lipid polymer nanocarriers induced a synergistic effect in inhibiting the cell growth of human lymphoblast B Raji [164].

Anticancer activity against Dalton’s lymphoma cells has been demonstrated by producing silver–selenium nanoparticles (Ag-Se) with QUE and gallic acid [165]. Furthermore, some studies have provided evidence that Nab-P (Abraxane^®^) non-covalently coated with rituximab (AR160) improved tumor efficacy due to increased antibody-mediated drug deposition in a human B-cell lymphoma and mouse Daudi cells [166]. These data represented the basis for the clinical development of AR160, which is currently in progress [167]. All these results strongly suggest that integrating nanoformulation with phytocompounds has widely shown synergistic effects, enhancing therapeutic efficacy against lymphoma while minimizing adverse effects. Further research is needed to optimize the formulations and validate their clinical efficacy.

**Table 6:** Phytocompound-based nanoformulations (NF) and their main advantages in lymphoma.

Phytocompounds	NF	Advantages	Refs.
Curcumin	Solid lipid NPs (SLN) d-α-tocopheryl polyethylene glycol 1000 succinate (TPGS)	Enhanced cytotoxic effect	[158]
		Reduction of proinflammatory cytokines	
		Enhanced apoptosis	
		Reduced tumor growth	
	mPEG-g-P (Glu-co-Phe)	Enhanced antitumor effect Enhanced apoptotic pathway Reduced invasive ability	[159]
	CUR/imatinib/NLCs	Enhanced therapeutic efficacy	[160]
		Increased tumor targeting	
		Increased cellular uptake	
Resveratrol	DOX/Resv/polymeric micelles	Enhanced therapeutic efficacy	[161]
Quercetin	QUE/VCR/lipid polymer	Enhanced antitumor effect	[164]
	QUE/gallic acid/Ag-Se	Enhanced antitumor effect Enhanced apoptosis	[165]
Paclitaxel	Nab-paclitaxel NPs (ABX)/rituximab (AR160)	Enhanced therapeutic efficacy	[166]
		Improved tumor targeting	
		Improved biodistribution	

### 2.4. Malignant Melanoma

Malignant melanoma (MM) is one of the most aggressive forms of skin cancer and is responsible for a significant proportion of cancer-related deaths worldwide. This type of cancer results from the oncogenic mutations (BRAF/NRAS/KIT) of melanocytes in the skin (cutaneous MM) and sometimes from non-dermal melanocytes. Melanocytes are pigment-producing cells derived from a neural crest located in the basal layer of the epidermis [168,169,170].

The increase in MM diagnosed in more developed countries is mainly due to exposure to ultraviolet light from sunlight, which represents the main risk factor for cutaneous MM development [171,172]; indeed, over the last 10 years, the number of MM cases has increased by 1.5% in the United States only [173,174].

The highly invasive and metastatic MM can develop a resistance phenotypic response to chemotherapy and radiotherapy, leading to a low survival rate in MM patients [175]. For this reason, MM is one of the most difficult tumors to fight with conventional therapies, including surgical excision, chemo-, radio-, and immunotherapies, gene therapy, and vaccines. Moreover, MM conventional cancer therapies cause undesiderable side effects. Hence, also for MM, in recent years, there has been growing interest from researchers in developing more-effective and less-toxic therapeutic strategies [176]. As mentioned above, phytocompounds can improve drug efficacy, reducing conventional drug adverse effects as well as sensitizing cancer cells to chemotherapeutic agents (see Table 7) [177].

#### 2.4.1. Phytocompounds

Resv showed chemosensitizing properties and chemopreventive activity in a wide range of cancers such as leukemia, carcinoma, breast, colon carcinoma, and MM [98,178,179,180]. Gatouillat et al. demonstrated that Resv was able to modulate the tumor suppressor gene p53 in chemoresistant B16 MM cells, inducing G1 cell cycle arrest and cell proliferation block. Moreover, Resv enhanced doxorubicin-induced cytotoxicity along with cyclin D1 downregulation [181]. In another in vitro study, Resv caused a cell growth decrease in A375 cells, inducing reactive oxygen species (ROS) generation, endoplasmic reticulum stress, and cell cycle arrest [182]. Larrosa et al. showed that Resv and the related molecule 4-hydroxystilbene induced growth inhibition, apoptosis, S-phase arrest, and upregulation of cyclins A, E, and B1 in human SK-Mel-28 MM cells [98,183]. A number of in vivo studies clearly demonstrated that Resv can delay tumor growth in several cancer types. Caltagirone et al. studied the effects of Resv on the growth and metastatic potential of B16-BL6 MM cells in vivo. After the simultaneous intraperitoneal administration of Resv and the intramuscular injection of B16 cells into syngenic mice, a dose-dependent tumor growth delay and the absence of systemic toxicity were observed [184].

The anti-angiogenic, pro-apoptotic, antiproliferative, and immunomodulatory properties of CUR were widely demonstrated in several human MM cell lines [185]. At the molecular and cellular level, CUR was able to slow down MM progression by influencing several molecules, such as Bcl2, MAPKS, p21, and some microRNAs, as demonstrated by several in vitro, in vivo, and even clinical studies [186,187,188,189,190,191,192]. Zhao et al. demonstrate that CUR was able to induce a block of cell invasion, cell cycle arrest, and autophagy in A375 and C8161 MM cells [193]. Moreover, preclinical animal experiments and phase I clinical studies shown that CUR induced low toxicity even at high doses (12 g/day), suggesting that CUR could be considered as a new therapeutic candidate for the management of MM [185,193,194,195,196].

QUE, a bioactive flavonoid found in fruits and vegetables such as apples, red grapes, and onions, shows multiple antiproliferative and anticancer properties [197]. It has been widely demonstrated to induce cell viability reduction, apoptosis, and autophagy, and to prevent metastasis by reducing VEGF secretion and MMP levels. Also, QUE is involved in different metabolic pathway modulation by inhibiting key enzymes of glycolysis, glucose uptake, and mitochondrial functionality, contributing to its final effects: metastasis inhibition and apoptosis/autophagy induction in cancer cells [198]. Sturza et al. demonstrated that QUE was able to inhibit ATP production at the mitochondrial level in murine MM cells (B164A5), causing tumor cell death [199]. In a study by Peng et al., QUE inhibited mouse MM growth in vivo by suppressing tumor proliferation and promoting apoptosis. In addition, the inhibition effect on migration and invasion in B16 and A375 cells was correlated with RIG-I promoter to induce IFN-I production [200].

Aloe is a medicinal plant with various pharmacological activities; it can contain molecules with important biological and toxicological functions, including flavonoids, vitamins, alkaloids, simple and complex polysaccharides, minerals, enzymes, and hydrocarbons. Aloe flavonoids exhibit anti-inflammatory, antioxidant, antimicrobial, and anti-aging properties and are used in the treatment and prevention of chronic diseases such as diabetes and cancer [201]. Several studies have reported the antitumor activity exerted by Aloe in MM ascribed to its nearly 75 active compounds characterized by high therapeutic value [202]. Aloe emodin is an anthraquinone extracted from Aloe that possesses most of the therapeutic properties attributed to the Aloe plant. Tabolacci et al. demonstrated that Aloe emodin was able to inhibit the metastasic properties of B16-F10 cells in vitro [203]. In another study, Aloe emodin induced cell proliferation reduction and differentiation in SK-MEL-28 and A375 melanoma cells. In addition, Aloe emodin treatment caused inhibition of cell metastatic ability and reduced the proliferation, stemness, and invasive potential of melanospheres, suggesting its potential employment in chemotherapy strategies also directed against resistant MM [204].

Essential oils (EOs) are low-molecular-weight volatile chemical compounds characterized by a strong odor deriving from the plant secondary metabolism. EOs can be extracted from all parts of plants, such as leaves, stems, flowers, seeds, roots, and bark. They are mainly constituted by monoterpenes, sesquiterpenes, and their oxygenated derivatives and are usually obtained via hydrodistillation or steam distillation [205]. Several studies demonstrated that EOs exert antitumor activity against skin cancer, including MM, through in vitro cell proliferation reduction, cell cycle alteration, apoptosis induction, in vitro inhibition of cell invasion and migration, in vivo tumor growth, metastasis, and angiogenesis decrease [206]. Ramadan et al. reported that tea tree oil (TTO), an essential oil extracted from *Melaleuca alternifolia*, induced apoptosis in A375 MM cells through caspases 3, 7, and 9 activation, upregulation of p53 and Bax proapoptotic proteins, and downregulation of bcl-2 [207]. Furthermore, in another study by Calcabrini et al., TTO and its main active component, terpinen-4-ol, were capable of inducing apoptosis in both sensitive and doxorubicin-resistant M14 melanoma cells. TTO interaction with the plasma membrane and the subsequent reorganization of lipid architecture were identified as a possible mechanism of caspase-dependent apoptosis induction [208,209]. Furthermore, Di Martile et al. demonstrated that TTO, used in combination with dabrafenib/trametenib, synergistically induced cell viability reduction correlated to apoptosis induction (caspase 3 activation, PARP cleavage) along with P-glycoprotein inhibition in MM models [210]. Moreover, they showed that three specific components of TTO (α-terpineol, tepinolene, and terpinen-4-ol) were responsible for MM antitumor action.

Among other terpenes with known anticancer properties, limonene (a monocyclic monoterpene found in citrus fruits) was reported to inhibit cell proliferation and to induce apoptosis in MM cells in both in vitro and in vivo studies [176]. Lupeol (a pentacyclic triterpenoid found in a variety of plants, including Mango, *Acacia visco*, *Camellia japonica*), with known anticancer and anti-inflammatory properties, suppressed the growth of several MM cell lines (Mel-928, Mel-1241, Mel-1011) by effectively blocking the Wnt/β-catenin signaling pathway [211]. As widely known, immunotherapy represents an effective treatment strategy for MM allowing for recurrence risk reduction and improvement of patient survival [212]. Unfortunately, not all patients can undergo this therapy, and most of them still present tumor recurrence. Recently, a number of phytocompounds (flavonoids, polysaccharides, terpenes among others) have been demonstrated to improve immunotherapy efficacy thanks to their ability to remodel the tumor microenvironment involved in cancer and their ability to escape from immune system recognition [213]. Among terpene compounds, β-caryophyllene was reported to enhance the antitumor activity of anti-PD-1 immunotherapy in an MM mouse model [214].

The pentacyclic triterpenoid BA, extracted from the bark of plane trees and birches, exerts antitumor activities against MM. Wróblewska-Łuczka et al. showed a significant inhibition of the in vitro growth of several MM cell lines, along with the antiproliferative activity of BA in combination with paclitaxel or docetaxel [215,216].

Ursolic acid (UA) is a pharmacologically active pentacyclic triterpenoid derived from the pomace, cork, flowers, buds, leaves, and bark of several medicinal plants. UA has multiple biological activities, such as antioxidant, anti-inflammatory, and anticancer activities, being involved in many pathways controlling proliferation and apoptosis. Unfortunately, UA exhibits poor bioavailability and absorption; for this reason, the original skeleton of the acid has been modified, and synthetic derivatives showing increased therapeutic effects have been developed [217,218]. Mahmoudi et al. demonstrated that treatment of human MM cells with UA resulted in the induction apoptosis through caspase activation [219].

THC and CBD have been largely studied for their potential therapeutic effects also in MM. Several studies have shown that these cannabinoids can inhibit cell proliferation, induce apoptosis, and suppress tumor angiogenesis [220].

Simmerman et al. demonstrated that CBD was able to reduce tumor growth and improve the quality of life and survival of C57BL/6 mice with MM induced by inoculation of the B16F10 cell line [221]. The anti-cancer effects of CBs in MM raise the possibility of using these compounds as adjuvants in its clinical management. Combining cannabinoids with conventional treatments, such as chemotherapy or immunotherapy, may enhance treatment efficacy and overcome resistance mechanisms [59].

Camptothecin (CPT) is a natural quinoline alkaloid isolated from *Camptotheca acuminata*, a tree native from Tibet and China. CPT shows anticancer properties deriving from DNA topoisomerase I inhibition, leading to DNA strand breaks and induction of apoptosis. In a study by Rudolf et al. CPT induced cellular stress responses in Bowes melanoma cells, including activation of p53-dependent DNA damage, mitochondrial- and caspase-dependent apoptosis, and p53-independent response to cellular stress [222].

**Table 7:** Phytocompounds and their main effects in melanoma.

Phytocompounds	Source	Antitumor Mechanism	Refs.
Resveratrol 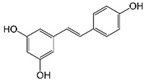	Grapes, blueberries, raspberries, mulberries, peanuts	↓ cell proliferation	[98,178,181,182,183]
		cell cycle arrest	
		↑ apoptosis	
		Modulation of p53 gene	[181]
		↑ doxorubicin-induced cytotoxicity	
		↓ cyclin D1	
		↑ ROS, endoplasmic reticulum stress	[182]
		↑ cyclins A, E, and B1	[183]
Curcumin 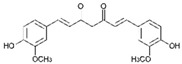	*Curcuma longa*	Immunomodulation	[185]
		↑ apoptosis	
		↓ proliferation	
		Regulation of Bcl2, MAPKS, p21and microRNAs	[186,187,188,189,190,191,192]
		↓ cell invasion	[193]
		↑ cell cycle arrest	
		↑ autophagy	
Quercetin 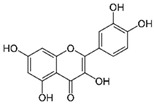	Citrus fruits, apples, onions, parsley, sage, tea, red wine	↓ cell viability	[198,199,200]
		↑ apoptosis	
		↑ autophagy	
		↓ metastasis	[198]
		↓ VEGF secretion and MMP levels	
		↓ enzymes of glycolysis	
		↓ glucose uptake	
		↓ mitochondrial functionality	
		↓ ATP production at mitochondrial level	[199]
		↓ migration	[200]
		↑ RIG-I promoter	
		↑ IFN-I production	
Aloe emodin 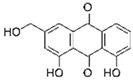	Aloe	↓ metastasis	[203,204]
		↓ cell proliferation	[204]
		↑ differentiation	
		↓ stemness	
		↓ invasive potential	
Tea Tree Oil	*Melaleuca alternifolia*	↑ apoptosis	[207,208,209,210]
		↑ caspases 3, 7 and 9	[207]
		↑ p53 and Bax	
		↓ bcl-2	
		Reorganization of lipid architecture	[208,209]
		↓ P-glycoprotein expression	[210]
		PARP cleavage,	
		↑ caspases 3, 7 and 9 levels	
Limonene 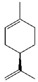	Citrus fruits	↑ apoptosis	[176]
		↓ cell proliferation	
Lupeol 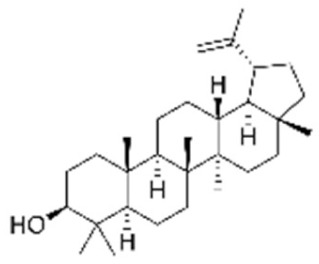	Mango, *Acacia visco*, *Camellia japonica*	↓ inflammation	[211]
		↓ cell proliferation	
		↓ Wnt/β-catenin signaling pathway	
Betulinic acid 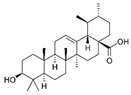	Birch, eucalyptus, plane trees	↓ cell proliferation	[215,216]
Ursolic acid 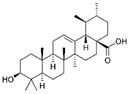	Apples, bilberries, cranberries, peppermint, lavender, oregano, thyme, prunes	↑ cell apoptosis caspase activation	[219]
Cannabinoids 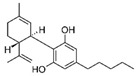	*Cannabis sativa*	↓ tumor growth ↑ survival	[221]
Camptothecin 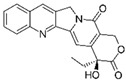	*Camptotheca acuminata*	↓ DNA topoisomerase I	[222]
		↑ p53-dependent DNA-damage	
		↑ mitochondrial and caspase-dependent apoptosis	
		↑ p53-independent response to cellular stress	

#### 2.4.2. Nanoformulations

As part of the employment of nanoformulations for natural products transport and delivery in MM (see Table 8), in a study by Carletto et al., Resv loaded into nanocapsules of polysorbate and administered to mice via MM xenograft was able to inhibit tumor growth efficiently [223].

Singh et al. demonstrated that nanoformulated CUR enhanced natural product cellular uptake and cytotoxicity effects compared to free CUR [224]. Co-delivery of CUR and STAT3 siRNA using cationic liposomes into female C57BL/6 mice induced a significant inhibition of tumor progression after topical and intrathecal administrations [225]. Also, CUR loaded into chitosan-coated poly-caprolactone nanoparticles and orally administered to mice (3 or 6 mg/kg) caused a reduction in MM lung metastasis appearance [226]. Finally, Lu et al. use CUR PEG-micelles, conjugated with a vaccine, to remodel the tumor microenvironment and enhance vaccine activity in an advanced MM model [227].

Dora et al. studied the in vitro and in vivo activity of QUE-loaded nanosized emulsion, demonstrating its ability to inhibit the proliferation of B16-F10 cells and tumor growth in C57BL76 mice after oral administration. Moreover, this nanoformulation did not induce renal or hepatic toxicity when compared to free QUE [228].

PLGA NP-loaded UA induced a higher cytotoxic effect and increased cellular uptake compared to the free compound in a study of Baishya et al. [229].

Aloe and its derivatives have been encapsulated into various carbon-based nanoformulations and then used in combination with antineoplastic drugs against different tumors, including MM, showing very promising synergistic effects [202].

Danciu et al. reported that silver-NP-loaded BA caused higher cytotoxicity and antiproliferative effects against both MM cell lines and MM mouse xenograft compared to free BA. Furthermore, encapsulated BA was able to reduce the metastatic ability in the vivo model [230].

Alipanah et al. analyzed the anticancer effects of chitosan NPs containing limonene and limonene-rich essential oils (*C. aurantium*, *C. limon*, and *C. sinensis*) in human BC and MM cell lines. They demonstrated that chitosan NPs containing *Citrus sinensis* and *Citrus limon* essential oils were the most effective against melanoma cell lines, suggesting that these nanoformulations can be further evaluated for anticancer applications [231].

To overcome THC and CBD poor solubility and bioavailability and allow for a faster CBs clinical translation, a number of studies analyzed the employment of different nanoformulations. Freire et al. encapsulated cannabis extract-CN in poly(thioether-ester)-PTEe nanoparticles and demonstrated that encapsulation was able to preserve the ability of CN to promote apoptotic death in B16F10 melanoma cells, also inducing AKT phosphorylation and LC-3 II accumulation. The encapsulation method was shown to be effective in achieving CN delivery or in cannabinoid-based anticancer therapies [232].

The clinical use of CPT is limited by its poor solubility in water and the instability of the active form in physiological media [233]. Lin et al. loaded CPT into liposomes coated with the α-melanocyte stimulating hormone (MSH) for targeted therapy against the melanoma B16-F10 cell line. Thanks to the load and transport by liposomes, a higher efficiency in cellular uptake and the subsequent cytotoxic effect were detected in melanoma cells as compared to free CPT. The liposomal functionalization with α-MSH could represent a promising approach to overcoming melanoma drug resistance and to reducing the negative side effects, minimizing the distribution to normal tissues [234].

In a study by Hu et al., a tumor grown in nude mice was treated with PEG-conjugated CPT and CUR to form the co-delivery system for synergistic inhibition of B16 MM cells. The results show that the CPT/CUR co-administration system induced a higher inhibition effect as compared to each agent alone, suggesting a synergistic therapeutic effect [235].

**Table 8:** Phytocompound-based nanoformulations (NF) and their main advantages in melanoma.

Phytocompounds	NF	Advantages	Refs.
Resveratrol	Polisorbate nanocapsules	Enhanced antitumor effect Decreased tumor size Increased necrotic area Increased inflammatory infiltrate Metastasis and pulmonary Hemorrhage inhibition	[223]
Curcumin	Cationic liposomes	Enhanced uptake Enhanced cytotoxicity	[225]
	Chitosan NPs	Enhanced antitumor effect MM lung metastasis reduction	[226]
	PEG micelles	Enhanced vaccine activity Downregulated immunosuppressive factor levels Increased proinflammatory cytokines levels	[227]
Quercetin	Nanoemulsions	Improved oral bioavailability Inhibited cellular proliferation and tumor growth	[228]
Ursolic acid	PLGA NPs	Increased uptake Slower blood clearance Increased antitumor effect	[229]
Aloe emodin	Carbon NF	Increased synergistic effect Enhanced antineoplastic effect	[202]
Betulinic acid	Silver nanocapsules	Increased antiproliferative effect Reduced secondary tumors development	[230]
Limonene	Chitosan NPs	Enhanced antitumor effect	[231]
Cannabinoids	PTE NPs	Dnhanced antitumor effect	[232]
Camptothecin	Liposomes	Higher antitumor activity Enhanced MDR-overcoming activity	[233]
CPT-CUR	Micelles	Increased synergistic effect	[235]

### 2.5. Glioblastoma Multiforme

The major form of primary human brain tumors is constituted by gliomas, classified as low-grade gliomas, glioblastomas, or anaplastic astrocytomas based on the degree of invasiveness and pathology of the tumor [236]. Glioblastoma multiforme (GBM) is the most malignant form of astrocytoma, representing one of the most aggressive tumors affecting the central nervous system.

GBM surgical resection, radiotherapy, and adjuvant chemotherapy represent the main treatment options [237]. Among chemotherapy drugs, TMZ, lomustine, dacarbazine, vincristine, cisplatin, carmustine, nimustine, and carboplatin are currently used for GBM treatment [237,238]. The heterogeneity of GBM, the presence of the blood–brain barrier (BBB), the infiltration ability into the adjacent healthy tissues, and the onset of multidrug resistance are responsible for GBM treatment difficulties and chemotherapy failure [239,240,241].

#### 2.5.1. Phytocompounds

In this context, a better understanding of GBM resistance mechanisms may lead to the development of new therapeutic strategies based on the employment of new potential molecules with therapeutic action against GBM. In this scenario, several phytocompounds, including soy isoflavones, CUR, EGCG, Resv, cannabinoids, and retinoids, have been demonstrated as promising pharmacologic tools against GBM (see Table 9) [242,243,244,245,246].

QUE, a flavonoid found in several plants and foods such as apples, onions, red grapes, cherries, honey, and green leafy vegetables, has been shown to exert antioxidant, antiviral, and anticancer properties by modulating the cell cycle, inducing apoptosis, and inhibiting angiogenesis in different types of cancer, including GBM [247,248]. This natural product induced apoptotic cell death in p53 mutant glioblastoma U373MG cells by decreasing, in a dose-dependent manner, the mitochondrial membrane potential and causing nuclear fragmentation [249]. As is known, low survival of GBM patients is associated with high expression of interleukin-6 (IL-6) [250,251]. QUE has been demonstrated to decrease IL-6 levels in a dose-dependent mode through STAT3 activation in T98G and U87 glioblastoma cells [252]. Moreover, QUE was able to sensitize two glioblastoma cells lines (U251 and U87) to TMZ treatment by inhibiting the expression of heat shock protein 27 [253].

In recent years, the non-flavonoid polyphenol Resv has attracted substantial attention due to its antioxidant and anti-inflammatory properties for treating ischemia and hypoxia due to its neuroprotective effects in penetrating the blood–brain barrier [254,255]. A number of studies revealed its ability to target different signaling pathways involved in cancer development and progression, resulting in apoptosis, autophagy, and senescence induction in GBM cells; moreover, Resv employment, both in single and combination therapies, achieved the re-sensitization of cancer cells to radiotherapy and induced chemosensitizing effects [256]. In combination treatments, Resv has been demonstrated to enhance TMZ toxicity in several GBM cell lines by inhibiting TMZ-induced G_2_/M arrest via the induction of the senescence pathway [257]. A study by Cilibrasi et al. has also demonstrated the effects of Resv in seven glioma stem cell (GSC) lines derived from GBM patients. By modulating the Wnt signaling pathway, Resv was able to inhibit the cell proliferation by increasing cell mortality and reducing motility of the cells [258,259,260]. Furthermore, by regulating the activation of NF-κB in U373MG glioma cells, Resv decreased the TNF-α-induced invasion [261]. In addition, Huang et al. have demonstrated Resv’s ability to modulate TMZ resistance through the downregulation of MGMT expression [262] by increasing the induction of apoptosis in TMZ-resistant T98G cells. These results strongly suggested that Resv may be used as an effective adjuvant molecule in GBM combination therapies.

Among bioactive phytocompounds with anti-GBM activities, polydatin, a stilbenoid polyphenol and a glucoside derivative of Resv, found in such plant families Vitaceae, Liliaceae, and Leguminosae, as well as in red wine, nuts, vegetables, and fruits, represents a promising molecule [263]. Compared to Resv, polydatin shows a higher anti-inflammatory [264] and antioxidant activity [265]. Polydatin treatment efficacy was demonstrated in different GBM cell lines by reducing cell proliferation, migration, invasion, and stemness and inducing apoptosis through the inhibition of EGFR-AKT/ERK1/2/STAT3-SOX2/Snail signaling pathway. In addition, PD showed no or very low cytotoxicity to normal human cells [266].

Numerous literature data suggest that CUR is involved in the modulation of most GBM signaling pathways [267]. CUR potential therapeutic and protective activities have been demonstrated against malignant tumors in the central nervous system thanks both to CUR’s ability to penetrate the BBB and to its lipophilic nature [268].

GBM cell’s ability to penetrate normal brain tissue is related to the very high expression of membrane-associated or secreted matrix metalloproteinases (MMPs) involved in extracellular matrix degradation. By inhibiting AP-1 and MAP molecules, CUR was able to suppress the expression of MMP-1, -3, -9, and -14 in GBM cell lines [267,269]. Wang and Chen reported that CUR affected different steps of angiogenesis process by suppressing the expression of transcription factors, NF-κB, and pro-angiogenesis factors (VEGF and βFGF) [270]. Moreover, Dhandapani et al. revealed that CUR reduced cell proliferation, inducing DNA fragmentation and apoptosis through a caspase-dependent pathway [271] and both increasing the BAX/Bcl-2 ratio and stimulating caspase 8 activation. CUR in combination with TMZ seemed to induce an additive cytotoxic effect in GBM cells by causing cell cycle block in the G_2_/M phase [272]. In addition, CUR-TMZ combination was able to induce ERK1/2-dependent autophagy [267]. Moreover, it has been reported that CUR enhanced the action of other numerous drugs, such as cisplatin, CPT, PTX, and doxorubicin, in different human GBM cell lines, leading to a synergistic effect [269,273]. Furthermore, CUR may prevent chemoresistance in GBM cells by reducing the expression of different ABC transporters [274]. Finally, as demonstrated by Trotta et al., CUR was able to sensitize GBM cells resistant to TRAIL therapy via apoptosis [275].

As described above, PTX is currently used for the treatment of several cancer types, including glioblastomas [276]. This natural compound was able to trigger apoptosis via the upregulation of the caspase signal pathway and affected U251 and U87MG cell growth and proliferation through MMP-9 and p38/JNK pathway inhibition [277]. Unfortunately, PTX activity against brain tumors was unsatisfactory in phase II experiments due to the presence of the BBB and the activity of drug-transporter proteins in both in vitro and in vivo conditions [278,279].

In numerous preclinical glioblastoma models, cannabinoids (CBD, THC) have demonstrated anticancer activities via the inhibition of cell proliferation associated with cancer cell death, along with evident effects on angiogenesis [280]. It is also necessary to mention that CB1 and CB2 receptors are expressed in GBM human cells. Twelves et al. reported that submaximal doses of THC and CBD in combination with TMZ treatment had high anticancer activity both in TMZ-sensitive and TMZ-resistant GMB tumors. In addition, the authors have reported preliminary results on the efficacy of nabiximols oro-mucosal cannabinoid spray used in combination with TMZ in GBM patients [281].

Betulin and its derivatives are phytocompounds of great interest. In particular, BA (3-beta-hydroxy-lup20(29)-en-28-oic acid), a derived pentacyclic lupine-type triterpenoid, has been demonstrated to possess different biological activities, such as antioxidants, anti-inflammatory, and anti-GBM action, via the downregulation of NF-kB, the suppression of pro-survival transcription factor Sp1, and the enhancement of TMZ cytotoxic effects [282,283].

**Table 9:** Phytocompounds and their main effects in glioblastoma multiforme.

Phytocompounds	Source	Antitumor Mechanism	Refs.
Quercetin 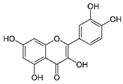	Citrus fruits, apples, onions, parsley, sage, tea, red wine	Cell cycle modulation	[247,248,249]
		↑ apoptosis	
		↓ angiogenesis	
		↓ mitochondrial membrane potential	[249]
		nuclear fragmentation	
		↓ IL-6 levels	[252]
		↑ STAT3	
		↓ heat shock protein 27 expression	[253]
Resveratrol 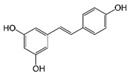	Grapes, blueberries, raspberries, mulberries, peanuts	↑ apoptosis	[256]
		↑ autophagy	
		senescence induction	
		chemo-sensitization	
		Wnt signaling pathway modulation	[258,259,260]
		↓ cell proliferation	
		↓ cell mortality	
		↓ cell motility	
		↑ NF-κB	[261]
		↓ TNF-α induced invasion	
		↓ MGMT expression	[262]
Polydatin 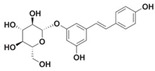	Grapes, blueberries, raspberries, mulberries, peanuts	↓ cell proliferation	[266]
		↓ migration and invasion	
		↓ stemness	
		↓ EGFR-AKT/ERK1/2/STAT3-SOX2/Snail signaling	
Curcumin 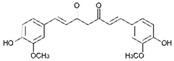	*Curcuma longa*	↓ MMP-1, -3, -9 and -14 expression	[267,269]
		↓ p38, JNK, ERK	
		↓ angiogenesis	[270]
		↓ NF-κB	
		↓ VEGF, βFGF and MMPs	
		↓ cell proliferation	[271]
		↑ DNA fragmentation	
		↑ apoptosis	
		↑ BAX/Bcl-2	[272]
		↑ caspase 8	
		↓ ABC transporters expression	[274]
		chemo-senzitization	[269,273,274]
Paclitaxel 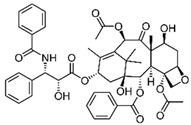	*Taxus brevifolia*	Chromosome mis-segregation	[276]
		Mitotic arrest	
		↑ apoptosis	[277]
		↑ caspase signal pathway	
		↓ cell growth and proliferation	
		MMP-9 and p38/JNK pathway	
Cannabinoids 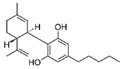	*Cannabis sativa*	↓ cell proliferation	[280]
Betulic acid 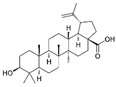	Birch, eucalyptus, plane trees	↓ NF-κB ↓ Sp1 ↑ cytotoxicity of TMZ	[282,283]

#### 2.5.2. Nanoformulations

As has been widely demonstrated, most therapeutic agents cannot adequately reach GBM due to the presence of the BBB. The hydrophobic nature of several chemotherapeutic drugs, their large dimensions, and the activity of MDR efflux pumps expressed both at the BBB level and in tumor cells represent key factors responsible for reduced drug accumulation in the brain. A number of nanocarrier-based DDS have been developed to achieve a specific transport system to cross the BBB (see Table 10) [284]. Several types of NPs able to diffuse into the brain and loaded with conventional anticancer drugs or phytocompounds have shown improved intracranial drug delivery [285]. To increase their selective action on GBM, the NP surface has been decorated and functionalized with specific receptors for BBB antigens [286]. As is known, exosomes possess the innate ability to cross through BBB [287]. They are nanoscale-sized vesicles secreted by living cells. Compared with other nanocarrier-based DDS, exosomes have some advantages like good biocompatibility and low immunogenicity. Recently, exosomes have been demonstrated to enhance QUE accumulation in the brain [288]. Heidarzadeh et al. reported that exosomes loaded with QUE and administered to rats increased the concentration of this natural compound in the brain up to 2.5-fold compared with free QUE [289]. In addition, the in vitro effect of QUE and the QUE-platelet system was evaluated in the U373-MG human glioblastoma cell line using natural human platelets as carriers for drug loading and delivery [290]. The authors demonstrated that QUE reached a threefold enhancement of solubility, followed by an increase in cytotoxic effects.

Many studies demonstrated the overcoming of poor Resv bioavailability through its encapsulation and controlled release using different nanocarriers [291]. As is known, transferrin receptors (TfRs) are upregulated in GBMs. Jhaveri et al. employed transferrin-modified polyethylene glycol liposomes (Tf-RES-L) as a Resv delivery system. By activating the caspase 3/7 pathway, functionalized Resv-loaded liposomes significantly increased apoptosis in GBM cells compared with the free drug [292]. Recently, another DDS for Resv was designed by Lin et al., who reported that Pep-1, a short peptide containing nine amino acids (CGEMG-WVRC), was able to bind to both subcutaneous and orthotopic GBM xenografts expressing IL-13Rα2, a 65 kD plasma membrane receptor, overexpressing in GBM tissues and able to mediate endocytosis after binding to its ligand. A Resv-loaded poly(ethylene glycol)-*b*-poly(caprolactone) (PEG-*b*-PCL; PP) system was synthesized and subsequently modified with Pep-1 (Pep-PP@Res). Then, the in vivo anti-glioblastoma efficacy of Pep-PP@Res was tested after systemic administration to nude mice bearing C6-formed subcutaneous transplanted tumors. At the end of the treatment, a 64.5% reduction of the C6 xenograft tumor volume was observed in the mice group treated with Pep-PP@Res in comparison with the control group. The tumor growth inhibition was clearly attributed to nanovector employment as free Resv-treated mice showed a tumor growth trend very similar to that of the untreated group. These results strongly suggest that the Pep-PP@Res system could have promising in vivo anti-glioblastoma activity [293].

Although earlier studies reported that polydatin and its metabolites can cross the BBB, some potential pharmacokinetic problems, such as low selectivity, rapid metabolism, and poor bioavailability, limit its clinical application [294]. Recently, it has been reported that in lipopolysaccharide (LPS)-treated neurons, polymeric nanovectors loaded with Polydatin showed inhibitory effects on both oxidative stress and inflammatory responses [295]. Moreover, polydatin-loaded liposomes could enhance the release profile of this natural product, improving its bioavailability and increasing drug circulation time [296,297]. Unfortunately, these data do not include studies on human glioblastomas tumor cell lines.

Recently, CUR-based delivery systems have shown potential in managing conditions in brain health. Luss et al. showed that amphiphilic poly(N-vi-vinylpyrrolidone) NPs may be a suitable CUR carrier (PVP-CUR NPs) for human glioblastoma treatment [298]. In another study, magnetic NPs were employed to co-deliver CUR and TMZ to 2D and 3D cultures of GBM T-98G cells. This nanoformulation was able to induce higher cytotoxic effect as compared to both single-drug-loaded and unloaded drugs [299]. In addition, the polylactic co-glycolic acid (PLGA) copolymer has been used as a safe biocompatible nano-drug delivery system, able to enhance the uptake of CUR by cancer cells through the EPR effect. Moreover, treatment of GBM cells with CUR-NPs led to oxidative stress marker reduction, including ROS, and a significant increase in SOD activity [300].

Several studies reported nanomedicine approaches to delivering PTX to GBM. Li et al. designed co-assembled ursolic acid NPs (UA NPs) embedded with PTX as drug delivery vehicles. The UA-PTX NPs were able to increase PTX water solubility, facilitating its passage across the BBB. In addition, the UA- PTX NPs enhanced the cytotoxic effects induced by PTX through inhibition of P-glycoprotein activity, underlining the promising potential of UA-PTX NPs for GBM treatment [301].

Wang et al. formulated solid lipid NPs (SLNs) loaded with PTX and naringenin, a flavonoid belonging to flavanone subclass, widely distributed in several citrus fruits, bergamot, and tomatoes [302]. The co-administration with naringenin allowed for the increase in the oral bioavailability and the anticancer effects induced by PTX. According to numerous reports, in comparison to free drug suspension, the cyclic RGD peptide-functionalized SLNs exhibited higher cytotoxicity against U87MG glioma cells [303]. Recently, the development of PTX-loaded polymeric NPs for brain tumor treatment was investigated. PLGA NPs containing PTX (216 nm particle size) were administered through intranasal and intravenous routes to rats. The results showed an evident accumulation of PTX in brain tissue. Unfortunately, the antiproliferative effect of the PLGA-PTX treatment on GBM cells was comparable to that of free PTX [304].

As previously reported, cannabinoids show low aqueous solubility and stability. In addition, they are susceptible to autoxidation. To overcome these problems, numerous colloidal carriers have been designed to increase their biocompatibility, bioavailability, and solubility [305]. Aparicio-Blanco et al. have demonstrated that a new promising tool for GBM therapy could be represented by CBD-loaded lipid nanocapsules (LNCs) characterized by cannabinoid encapsulation in the LNCs oil core. A prolonged drug release effect was achieved in vitro, suggesting that this system could decrease the required therapeutical administrations in clinical trials. Furthermore, LNCs was engineered with CBD to target GBM cells overexpressing cannabinoid receptors selectively. These CBD-decorated LNCs were able to reduce the IC_50_ values to a greater extent versus the antiproliferative activity induced by CBD-loaded LNCs [306]. The combination of both encapsulation and decoration strategies allowed us to obtain the greatest growth inhibitory effect on GBM cells, suggesting their potential in vivo employment.

Another promising drug delivery system for CBs in GBM therapy is represented by polymeric NPs obtained via biodegradable polymers [307]. Assadpour et al. reviewed the employment of different polymeric nanocarriers, including hybrid ones, for CBD encapsulation, aiming to enhance drug water solubility and passage across biological barriers [308].

In order to increase BA aqueous solubility and its ability to pass through the BBB, BA NPs were synthesized by the standard emulsion approach by Li et al. [309]. The results obtained have demonstrated that BA NPs induced a strong antiproliferative effect in an intracranial model of glioma through the downregulation of NF-κB and the activation of apoptosis. Finally, a recent work evaluated a BA delivery system through liposome encapsulation (BL); furthermore, to improve its antitumor targeting, the system was functionalized via the addition of multi-layer membranes derived from HeLa cancer cells (BLCM). MTT assays showed the increased anticancer effect induced by BLCM as compared to BL delivery system. In addition, in vitro immunofluorescence labeling experiments demonstrated the higher targeting ability against cancer cells exhibited by the BLCM system as compared to the BL one [310].

**Table 10:** Phytocompound-based nanoformulations (NF) and their main advantages in glioblastoma multiforme.

Phytocompounds	NF	Advantages	Refs.
Quercetin	Exosomes	Increased stability Increased uptake and accumulation	[288]
	Platelets	Improved solubility Enhanced antitumor effect	[290]
Resveratrol	Transerrin-functionalized liposomes	Increased stability Enhanced drug-loading ability Prolonged drug release Enhanced apoptosis Increased in vivo tumor growth inhibition	[292]
	Pep-PP@Res system	Increased in vivo tumor growth inhibition	[293]
Polydatin	Polymeric nanovectors	Reduced oxidative stress response Increased oral bioavailability Prolonged in vivo circulation time	[295,296,297]
Curcumin	Poly-N-vinylpyrrolidone NPS	Prolonged drug action Increased accumulation Enhanced cytotoxic effects	[298]
	Magnetic NPs	Increased apoptosis Enhanced synergistic effect (CUR/TMZ)	[299]
	Chitosane PLGA NPs	Increased uptake via EPR mechanism Reduced oxidative stress response	[300]
Paclitaxel	Ursolic acid NPs	Increased water solubility Improved BBB crossing Enhanced cytotoxicity Drug efflux protein inhibition	[301]
	SLN-NPS PTX-Naringenin	Increased in vitro drug release Improved in vivo drug absorption Increased cytotoxic effect	[302]
	PLGA nanoparticles	Improved drug controlled release Reduced systemic toxicity Increased intratumor accumulation	[304]
Cannabinoids	Colloidal carriers	Increased water solubility, stability, and bioavailability	[305]
	Lipid nanocapsules	Prolonged drug release Enhanced cell proliferation inhibition	[306]
	Polymeric nanocarriers	Improved bioavailability Enhanced controlled biodistribution/Targetability/therapeutic efficacy	[308]
Betulinic acid	BA self-assembled NPs	Enhanced cell proliferation inhibition Increased apoptosis Improved BBB crossing in vivo Inhibition of tumor growth	[309]
	BA liposomes	Improved water solubility and bioavailability Increased in vitro growth inhibition Improved targeting properties	[310]

### 2.6. Osteosarcoma

Osteosarcoma is the most common primary bone cancer, and it is characterized by the production of osteoid from the neoplastic cells.

It has an incidence of 3–4 cases per million every year [311,312], with a first peak during the age range of 15–19 years and a second peak in the seventh and eighth decades [313,314]. It demonstrates a high rate of morbidity and mortality due to its high malignancy, early metastasis, and easy drug resistance [315]. The primary sites are long bones, such as distal femur, proximal tibia, and proximal humerus. The current standard therapy includes surgery to remove the tumor and neo adjuvant chemotherapy (i.e., doxorubicin, methotrexate, and cisplatin) to preserve the functioning of the operated part and prevent recurrence and metastasis. This combined strategy increased the 5-year survival rate of patients to 60%, even if a substantial improvement of treatment has not been achieved in recent decades [316]. Moreover, prognosis remains extremely poor for 20% to 30% of patients regardless of the treatment taken [317]. The main limitation of chemotherapy is the side effects developed during a prolonged administration of large doses, causing the interruption of drug and, consequently, the progression of osteosarcoma. Therefore, there is an urgent need to develop new therapeutic strategies for osteosarcoma, adopting multidisciplinary approaches and new technologies. Among the new strategies, natural products and nanotechnology became the new frontiers for osteosarcoma treatment.

#### 2.6.1. Phytocompounds

Bioactive compounds derived from natural sources have been investigated for the improvement of the efficiency of osteosarcoma treatment. Evidence is currently available for the promising effects of several phytocompounds, including curcumin, genistein, resveratrol, berberine, quercetin, tanshinone, silibinin, and galangin [318]. Their main effects refer to the induction of apoptosis and autophagy; the inhibition of proliferation, growth, invasion, and migration; the reduction of motility and viability; the promotion of radiosensitivity; and cell cycle arrest. These effects are related to the capability of the compounds to modulate the signal pathways of osteosarcoma (see Table 11) [319]. Specifically, the major pathways are the Notch signaling pathway for CUR and DATS, the WNT/β-catenin signaling pathway for Resv and apigenin, and the Hedgehog PI3/AKT signaling pathway for cyclopamine and SFN [320]. Recently, decursin has also been investigated for its sensitizing effect in combination with cisplatin [321]. Decursin is a pyranocoumarin compound derived from the roots of *Angelica gigas Nakai*, a natural plant that grows in Southeast Asia and China. This compound is usually used to treat many disorders, such as anemia, dysmenorrhea, amenorrhea, and menopausal disorders. Many studies show that decursin has many biological activities, such as antioxidant, anti-inflammatory, neuroprotective, and anticancer activities [321,322,323]. Decursin was found to suppress cell viability and induce apoptosis via the cell cycle. Moreover, it can suppress the phosphorylation of AKT. The combined action with cisplatin can cause the induction of apoptosis, the reduction of cell viability, and decreased tumor volume in mice. In addition, they jointly have a function of renal protection with the reduction of renal epithelial cell damage [321].

**Table 11:** Phytocompounds and their main effects in osteosarcoma.

Phytocompounds	Source	Antitumor Mechanism	Refs.
Curcumin 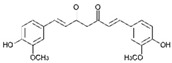	*Curcuma longa*	Notch signaling pathway modulation	[320]
Diallyl trisulfide 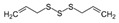	*Allium sativum*		
Resveratrol 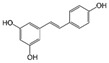	Grapes, blueberries, raspberries, mulberries, peanuts	WNT/β-catenin signaling pathway modulation	[320]
Apigenin 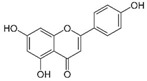	Parsley, onions, oranges, tea, chamomile, wheat sprouts		
Cyclopamine 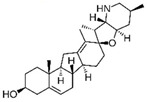	*Veratrum dahuricum*, *Veratrum grandiflorum*, *Veratrum californicum*	Hedgehog PI3/AKT signaling pathway modulation	[320]
Sulforaphane 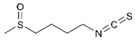	*Brassica oleracea*		
Decursin 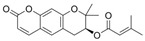	*Angelica gigas Nakai*	↓ cell viability	[321]
		↑ apoptosis	
		cell cycle modulation	
		↓ Akt phosphorylation	
Cannabidiol 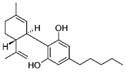	*Cannabis sativa*	↓ tumor growth	[324]
		↓ cell migration	

Also, cannabinoids were proven to exert an antiproliferative and anti-invasive activity against osteosarcoma. Specifically, CBD can act as an inhibitor of tumor growth and migration in osteosarcoma cells [324]. Although the majority of research has elucidated the effect of THC, its psychoactive effects may limit the application at vast scale [325]. Therefore, CBD application has been proposed as a substitute of THC due to the absence of the harmful effects whilst maintaining antitumor mechanisms against osteosarcoma. Some of the most recent evidence has demonstrated the synergistic inhibitory effect of doxorubicin/CBD on MG63 and U2R human osteosarcoma cell lines [326]. CBD and doxorubicin had a higher synergistic effect in the inhibition of osteosarcoma cells, compared with the single treatment alone, through the PI3K/AKT/mTOR and MAPK pathways. The co-treatment induced a higher inhibition of the invasion and migration of MG63, and U2R demonstrated a higher apoptosis and inhibition of cell cycle progression.

#### 2.6.2. Nanoformulations

Another strategy by which to enhance the therapeutic effect and reduce the toxicity of anticancer drugs in osteosarcoma treatment is provided by the application of nanotechnology. Liposome technology represents one of the most successful nanotechnologies applied to osteosarcoma treatment. Liposomes act as a vehicle by which to increase the delivery of the drug to the cancer site and, consequently, reduce the drug’s side effects [327]. Evidence has demonstrated the increased cell permeability and tumor cell death when liposomes are loaded with doxorubicin compared to free doxorubicin [328]. Similarly, the effect of the increased cell permeability and osteosarcoma cell killing was achieved by the application of the pH gradient method for the encapsulation of doxorubicin in the liposomes, with an 84% efficiency [329,330]. A new nanotechnology-based DDS was developed with the application of the keratin-based nanoformulation, demonstrating the improvement of treatment efficacy and prognosis in relapsed osteosarcoma patients [331]. Further approaches used PEGylated liposomes formulation for doxorubicin delivery during a Phase II Trial [332] and for siRNA delivery, alone and with doxorubicin, in the human MG-63 cell line [333] and human SaOs-2/MG-63 cell lines [334]. Evidence for the application of nanoformulation technology with a natural product emerged for CUR (see Table 12). CUR solubility and therapeutic efficacy are improved with the use of numerous encapsulation-based delivery systems. These systems include lipid nanomaterials (i.e., lipid particles, liposomal NPs), polymeric nanomaterials (i.e., amphiphilic NPS, poly NPs, polymerid NPs, micelles, hydrogels), inorganic nanomaterials (i.e., nanoarrays, nanofibrous 3D printed scaffolds), and metal nanomaterial. Therefore, the combination of CUR carriers and common neo-adjuvant chemotherapies has been proved to activate the expected molecular mechanisms of CUR in human osteosarcoma both in vitro and in vivo [335]. In particular, the mechanisms comprise an increase in apoptosis, autophagy, cytotoxicity, and tumor regression, along with a decrease in tumor size, cell viability, and proliferation.

In conclusion, a substantial body of literature proves the existence of strategies by which to counteract the negative effects induced by chemotherapy with the crucial contribution provided by phytocompounds and the application of nanotechnology. However, additional studies are required to explore natural bioactive compounds and their related signaling pathways further. With the advancement of nanomedicine, its application of nanotherapy in pediatric cancer will create new means of tackling osteosarcoma.

## 3. Conclusions

Natural products represent a significant source for developing compounds active in different diseases, such as cancer. Some pre-clinical and clinical studies have widely demonstrated the antitumor activities of several phytocompounds in various cancer types. Regrettably, their limited development and use as chemotherapeutic agents are due to various drawbacks. These include extract heterogeneity, toxicity against normal tissues/organs, low solubility, poor bioavailability, onset of resistance to treatments, and lack of target selectivity. Moreover, after administration, the chemical structure of phytocompounds can undergo severe changes due to their interaction with chemical and physical barriers, affecting their anticancer properties.

For several years now, nanotechnology has been a necessary tool for achieving different objectives related to natural compound safety and anticancer efficacy. These objectives include improving their cellular uptake, preserving their parental structure from environmental factors, increasing their aqueous solubility and bioavailability, and reducing their off-target effects, including risks of toxicity. Furthermore, nanotechnology has allowed for the encapsulation/transport of multiple substances (phytocompounds and conventional anticancer drugs) to perform combination treatments, overcoming drug resistance mechanisms and achieving a synergistic response. The employment of different approaches (receptor-mediated drug delivery, application of chemical or physical stimuli) has made it possible to direct the action of phytocompounds selectively, both alone and in combination, in space and time, allowing for a targeted delivery strategy.

Despite the significant results achieved in nanotechnology research, there are still challenges related to nanoformulation employment that significantly hinder its success in clinical settings. Some problems are linked to nanocarrier characteristics, such as particle size (which can induce NP clearance via the excretory system or sequestration via the mononuclear phagocytic system), high nanocarrier reactivity (due to some features such as charge, hydrophobicity, and the presence of surface functional groups responsible for nanocarrier interaction with host molecules/systems), the possible presence of a wide range of materials (including endotoxins arising from production process), whose safety and biocompatibility have not been widely tested in humans, and some potential host toxicities (allergic and hypersensitivity reactions, hemolysis, and thrombogenicity). Therefore, all these concerns must be considered from the early development steps of natural-product-based nanoformulations to enhance their safety and antitumor efficacy profiles to avoid subsequent problems in pre-clinical studies.

In addition, the long-term effects of nanocarrier employment are still not well studied and defined, necessitating further long-term toxicity studies before the start of clinical trials. Another important problem to be considered is related to nanoformulation feature reproducibility during the production process. Nanocarrier characteristics and/or drug loading procedures can undergo slight but significant variations, leading to further slowdowns or even negative outcomes during the preclinical evaluation process. Improved monitoring activities in each production step are therefore mandatory to guarantee the needed reproducibility. Finally, the choice of adequate experimental models mimicking human tumors (i.e., organ-on-chips, animals) is crucial to better evaluate nanoformulation pharmacokinetics and their efficacy and to accelerate their clinical translation.

Thus, although phytocompounds and their nanoformulations have been widely shown to have therapeutic advantages compared with conventional treatment strategies, there are still several problems associated with their rapid clinical translation, such as the complexity of multiple mechanisms exerted by most natural products, nanoformulation reproducibility and safety concerns, and sustainability problems linked to natural product finding, large-scale production, and cost challenges. Suitable experimental model employment, nanocarrier long-term toxicity and efficacy studies, the integration of innovative methods (molecular modeling, dynamic simulations, deep learning) to identify new phytocompounds with anticancer potential, better monitoring activities during nanoformulation development/production, and widespread public–private collaborations represent important instruments that could contribute to accelerating the nanoformulation translation process to clinical cancer therapy.

## Figures and Tables

**Table 12:** Phytocompound-based nanoformulations (NF) and their main effects in osteosarcoma.

Phytocompounds	NF		Advantages	Ref.
Curcumin	Lipid NPs		Increased water solubility and bioavailability Enhanced cytotoxic effect Higher cell cycle proteins expression Reduced tumor size	[335]
	Polimeric NPs		Increased stability and bioavailability Improved systemic circulation Improved targeting and delivery Enhanced antitumor effect Reduced stemness, migration, and invasion properties	
	Inorganic NPs		Increased bioavailability Improved targeting Enhanced cytotoxicity Higher anti-inflammatory activity	
	Metal NPs		Reduced cell proliferation Improved postoperative defect repair	
